# Single‐cell transcriptomic analysis in clear cell renal cell carcinoma: Deciphering the role of APP within the tumour microenvironment

**DOI:** 10.1111/jcmm.18186

**Published:** 2024-03-06

**Authors:** Bo Guan, Ming Li, Di Cui, Chen Shen, Zongyao Hao, Xiaowei Li

**Affiliations:** ^1^ Department of Urology Fuyang People's Hospital of Anhui Medical University Fuyang China; ^2^ Fuyang Medical College Fuyang Normal University Fuyang China; ^3^ Department of Urology Renji Hospital Shanghai China; ^4^ Department of Urology the First Affiliated Hospital of Anhui Medical University Anhui China; ^5^ Institute of Urology Anhui Medical University Anhui China; ^6^ Department of Nephrology Fuyang People's Hospital of Anhui Medical University Fuyang China

**Keywords:** amyloid precursor protein (APP), clear cell renal cell carcinoma (ccRCC), tumour microenvironment (TME), tumour‐associated macrophages (TAMs)

## Abstract

Clear cell renal cell carcinoma (ccRCC) represents a significant challenge in oncology, primarily due to its resistance to conventional therapies. Understanding the tumour microenvironment (TME) is crucial for developing new treatment strategies. This study focuses on the role of amyloid precursor protein (APP) in tumour‐associated macrophages (TAMs) within the ccRCC TME, exploring its potential as a prognostic biomarker. Basing TAM‐related genes, the prognostic model was important to constructed. Employing advanced single‐cell transcriptomic analysis, this research dissects the TME of ccRCC at an unprecedented cellular resolution. By isolating and examining the gene expression profiles of individual cells, particularly focusing on TAMs, the study investigates the expression levels of APP and their association with the clinical outcomes of ccRCC patients. The analysis reveals a significant correlation between the expression of APP in TAMs and patient prognosis in ccRCC. Patients with higher APP expression in TAMs showed differing clinical outcomes compared to those with lower expression. This finding suggests that APP could serve as a novel prognostic biomarker for ccRCC, providing insights into the disease progression and potential therapeutic targets. This study underscores the importance of single‐cell transcriptomics in understanding the complex dynamics of the TME in ccRCC. The correlation between APP expression in TAMs and patient prognosis highlights APP as a potential prognostic biomarker. However, further research is needed to validate these findings and explore the regulatory mechanisms and therapeutic implications of APP in ccRCC.

## 1 INTRODUCTION

Renal cell carcinoma (RCC) represents a significant oncological challenge, accounting for approximately 2% of global cancer diagnoses and deaths. Its incidence has more than doubled in the developed world over the past 50 years, making it the ninth most common neoplasm in the United States.^1^ RCC is notably the most prevalent solid lesion within the kidney, comprising about 90% of all kidney malignancies. This heterogeneous group of cancers arises from renal tubular epithelial cells, encompassing subtypes like clear cell RCC, papillary RCC and chromophobe RCC.^2^ The disease is characterized by a lack of early warning signs, variable clinical manifestations, resistance to radiation and chemotherapy and infrequent responses to immunotherapeutic agents.^3^

Clear cell renal cell carcinoma (ccRCC) is the most prevalent subpopulation of kidney cancer, characterized by its origin in the proximal uriniferous tubules and a tendency for aggressive behaviour, including metastasis to distant organs. ccRCC presents significant challenges in treatment, exhibiting high morbidity and mortality globally, and resistance to standard therapies like radiotherapy and chemotherapy.^4^ Current treatment modalities, primarily surgical intervention, show limited efficacy, highlighting the urgent need for reliable prognostic biomarkers to guide clinical decision‐making.^5^

Tumour‐associated macrophages (TAMs), a major component of the tumour microenvironment in ccRCC, have garnered attention for their dual role in both promoting and inhibiting cancer progression.^6^, ^7^ These macrophages are known to contribute significantly to the complexity of the tumour microenvironment, influencing tumour growth, metastasis and immune evasion.^8^ In ccRCC, the prognostic value of TAMs has been particularly noted; high densities of CD68^+^ TAMs and M2‐polarized TAMs are identified as risk factors for poor prognosis.^9^ Elevated levels of these TAMs correlate with worse overall survival (OS) and progression‐free survival (PFS), suggesting that TAMs could serve as potential biomarkers for prognosis and novel targets for immunotherapy in ccRCC.^10^, ^11^

In the context of molecular factors influencing ccRCC, amyloid precursor protein (APP) has emerged as a significant player. APP, historically associated with Alzheimer's disease, has recently been linked to the pathogenesis of various cancers, including ccRCC.^12^, ^13^, ^14^ It is involved in regulating cancer cell adhesion, a critical step in cancer metastasis and invasion. The pathological role of APP in malignancy, particularly in its potential molecular mechanisms related to cell proliferation and metastasis, has been explored in breast cancer, suggesting a similar exploration in RCC could yield valuable insights.^15^ Studies have shown that the expression of APP, along with EPB41L1 (a gene coding for a protein involved in cell adhesion), is downregulated in ccRCC, leading to poor prognosis.^16^, ^17^ This downregulation facilitates increased cancer cell metastasis and tumour invasion.^4^ Interestingly, the coordinated regulation of cancer cell adhesion by APP and EPB41L1 points to the possibility of these proteins as key factors in the progression of ccRCC.

This study aims to delve deeper into the role of APP within the ccRCC tumour microenvironment, particularly in relation to TAMs, and to explore its potential as a prognostic biomarker. By employing single‐cell transcriptomic analysis in conjunction with clinical data, we hope to uncover novel insights into the pathogenesis of ccRCC and identify new therapeutic targets, thereby improving clinical outcomes for patients with this challenging disease.

## 2 MATERIALS AND METHODS

### 2.1 Acquisition of single‐cell sequencing data

Single‐cell sequencing data (GSE152938) were obtained from the GEO database (https://www.ncbi.nlm.nih.gov/geo/). The dataset contains a total of five samples (two clear cell renal cell carcinoma samples, one papillary renal cell carcinoma sample, one chromophobe renal cell carcinoma sample and one normal kidney sample) from four patients. In addition, the renal clear cell carcinoma samples and normal kidney sample were used in our study. The raw data were presented in the form of standard 10X files, which was collated to generate Seurat objects for downstream analysis.

### 2.2 Acquisition of RNA sequencing and clinical data

The transcriptomic and clinical data of the study were obtained from the TCGA database (https://cancergenome.nih.gov/). The downloaded transcriptomic data were collated into a gene expression matrix, and a total of 614 patients with KIRC were further analysed. Finally, 534 patients with KIRC were included in the study after standardization of the above data and matching of clinical information. The rearranged gene expression matrix and raw clinical data were used for the next step of analysis.

### 2.3 Identification of TAM and immune landscape in KIRC

Analysis was performed using the Seurat R package. To remove overexpressed and over‐sequenced cells, the expression matrix was filtered using the following criteria: nCount_RNA > 1000 & nFeature_RNA < 5000 & percent.MT < 30 & nFeature_RNA > 600. Then, PCA reduction and UMAP reduction were used to cluster all cells, and the clustered cells were annotated with cluster marker genes. TAM was extracted for PCA reduction and UMAP reduction. Pseudotime analysis was performed using the monocle R package, which was used to explore the trajectory of gene changes in TAM. The BEAM function was used to explore the specific changes of genes in the TAM trajectory.

### 2.4 Construction of intercellular communication between tumour epithelia and TAM

Analysis was conducted using CellChat R package. The preprocessed scRNA‐seq data were input into the CellChat package. Initially, CellChat identified potential ligand–receptor pairs by referencing the comprehensive CellChatDB, a curated database of known and predicted cell–cell communication signals, including cytokines, growth factors and their corresponding receptors. We selected tumour epithelia and TAM as objects of intercellular communication analysis.

### 2.5 Construction of TAM‐related prognostic model in KIRC

To determine the association between tumour‐associated macrophage (TAM)‐related genes and patient prognosis, we conducted univariate and multivariate Cox proportional hazards analyses. We considered *p*‐values less than 0.01 as statistically significant. Following this, we undertook a secondary screening of the prognostic‐related genes using Kaplan–Meier analysis, through which we identified genes associated with TAMs that have prognostic significance. These genes were then used to develop a prognostic model, with risk scores computed using the following formula:

$$\text{Riskscore}=\sum_{i=0}^{n}\text{coef}\left(\text{RNAi}\right)\times \text{expr}\left(\text{RNAi}\right)$$

coef(RNAi) was defined as the coefficient of RNA corelated with survival. expr(RNAi) was defined as the expression of RNA.

Based on the median risk scores, patients with KIRC were categorized into high‐risk and low‐risk groups. To determine the survival differences between these groups, we employed the Kaplan–Meier survival analysis. All analyses were performed using the survival package in R.

### 2.6 TAM‐related gene prognostic model effect evaluation

We assessed the efficacy of risk scores by applying univariate and multivariate Cox regression analyses to examine the association between the predictive model and patient survival in KIRC. The time‐dependent receiver operating characteristic (ROC) curve analysed the model's predictive capacity, with the area under the curve (AUC) quantifying this measure. These analyses were conducted using the survival and survival ROC packages in R.

### 2.7 Acquisition of blood samples and tissue samples of KIRC

Blood samples were collected from 48 patients with renal clear cell carcinoma (RCCC) at the Department of Urology, Renji Hospital, stratified by disease stage: 18 with stage 1, 10 with stage 2, 10 with stage 3 and 10 with stage 4 RCCC (Table 1). Additionally, 30 blood samples from healthy individuals were acquired from the Renji Hospital Medical Examination Centre. For primary macrophage extraction, three RCCC tissue samples and one from resected renal malformation were procured from Renji Hospital's Department of Urology. Informed consent was obtained from all participants, and the study received approval from the Ethics Committee of Renji Hospital.

**TABLE 1:** Basic information of patients with RCCC.

Parameter	No. of patients (*n* = 48)
Age	59.98
Gender	
Male	33 (68.75%)
Female	15 (31.25%)
Stage	
I	18 (37.5%)
II	10 (20.83%)
III	10 (20.83%)
IV	10 (20.83%)
T stage	
1	18 (37.5%)
2	11 (22.92%)
3	16 (33.33%)
4	3 (6.25%)
N stage	
0	18 (37.50%)
1	5 (10.42%)
2	25 (52.08%)
M stage	
0	38 (79.17%)
1	10 (20.83%)

### 2.8 Fluorescence‐activated cell sorting and cell culture

Cell sorting was performed with a BD FACSAriaIII flow cytometer. Cell suspensions were stained with antibodies against CD11b (Invitrogen; CAS CD11B01), CD163 (Invitrogen; CAS 12‐1639‐42) and CD80 (Invitrogen; CAS 12‐0809‐42), following standard flow cytometric procedures. Post‐sorting, cells were cultured in T25 flasks (Servicebio; CAS CCF‐T25H) with RPMI 1640 medium (Corning; CAS 10‐040‐CV), supplemented with 10% fetal bovine serum (FBS) (Gibco; CAS C0235) and 0.05 mM β‐mercaptoethanol (Aladdin; CAS M301573). After 3 days, the cells were harvested into 50 mL centrifuge tubes (Servicebio; CAS EP‐5001‐J) and lysed with RIPA buffer (Beyotime; CAS P0013B) to extract proteins.

### 2.9 Enzyme‐linked immunosorbent assay

Plasma was isolated by centrifuging blood samples from 48 patients with clear cell carcinoma and 30 healthy individuals. APP levels in the plasma and primary macrophage were quantified using an APP‐human ELISA kit (Weiaobi; CAS EH10034M), following the manufacturer's protocol.

### 2.10 Tissue microarray

Our research engaged OUTDO BIOTECH to employ tissue microarrays for the investigation of APP alterations at the cancer tissue level. The firm conducted the experiments utilizing immunohistochemical antibodies against APP (Invitrogen; CAS 13‐0200) that we provided.

### 2.11 Statistical analysis

The R software (version 4.2.2) was used for all analyses. Functional annotation was performed using the Metascape database (https://metascape.org), and visualization was performed using the ggplot2 R package. Prism 9 was used for data analysis and survival curve plotting, and Student's *t*‐test and one‐way ANOVA were used to confirm statistical significance. *p* < 0.05 was considered statistically significant.

## 3 RESULTS

### 3.1 Sc‐RNA atlas of KIRC

**FIGURE 1:**
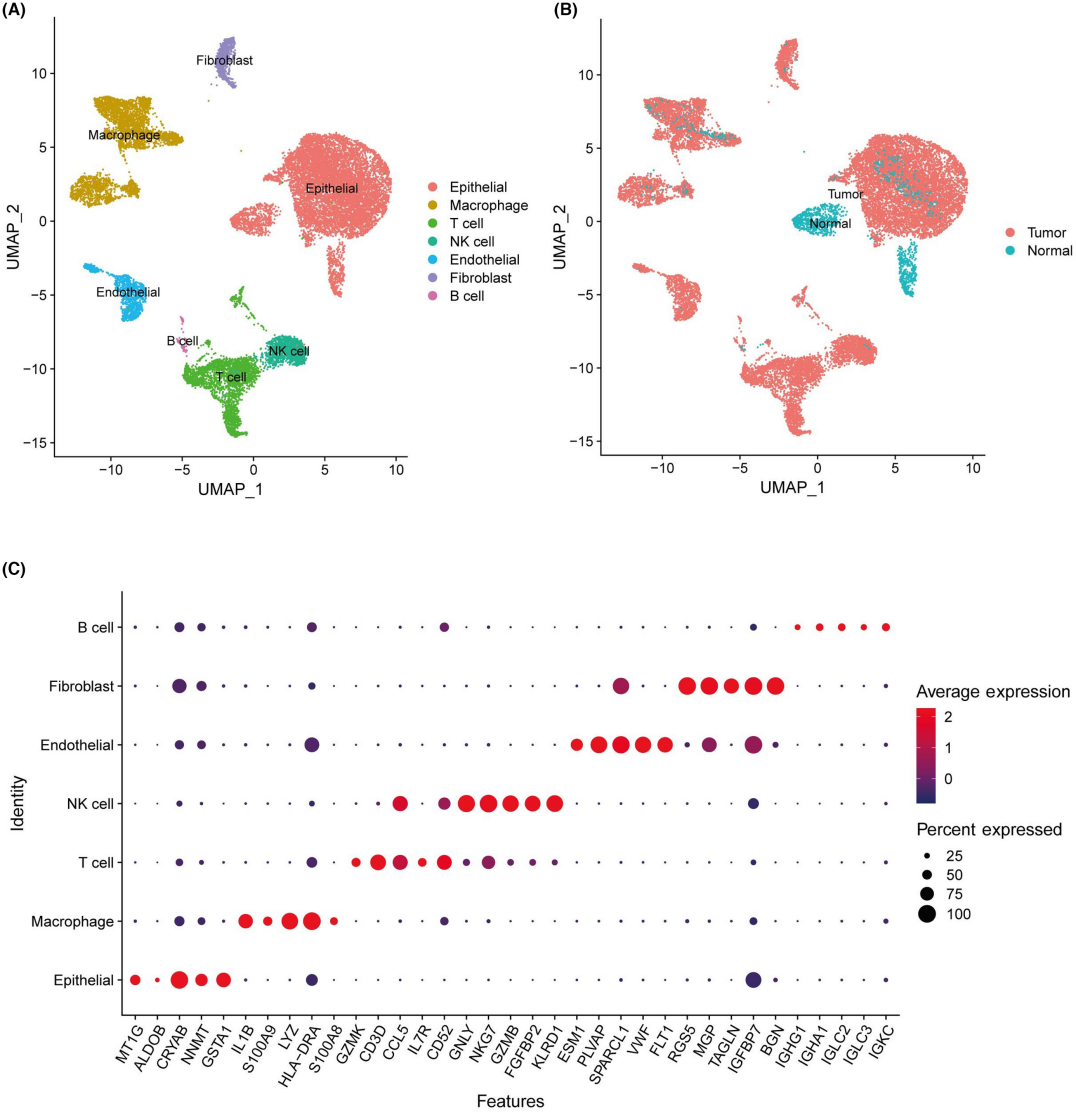
The atlas of ccRCC at single‐cell level resolution. (A) The cell type clusters of ccRCC. (B) The group clusters of ccRCC. (C) The marker genes of cell type clusters, and the red dot represents high level of average expression, while blue dot represents low level of average expression, besides, the size of dot represents percentage of cells expressing the marker.

We performed single‐cell RNA (sc‐RNA) sequencing on kidney renal clear cell Carcinoma (KIRC) samples, yielding 32,498 high‐quality cells for in‐depth analysis. Post‐UMAP (Uniform Manifold Approximation and Projection) dimensionality reduction, these cells clustered into 21 distinct groups annotated with specific marker genes as shown in Figure 1C. From these, seven cell types were identified via the CellMarker database (http://xteam.xbio.top/CellMarker/), predominantly immune cells (Figure 1A), highlighting their potential pivotal role in tumorigenesis. Notable heterogeneity was observed between KIRC and normal kidney tissues (Figure 1B), indicating diverse cellular functions and the establishment of a tumour microenvironment conducive to cancer development.

### 3.2 Identification of specific TAM of KIRC

**FIGURE 2:**
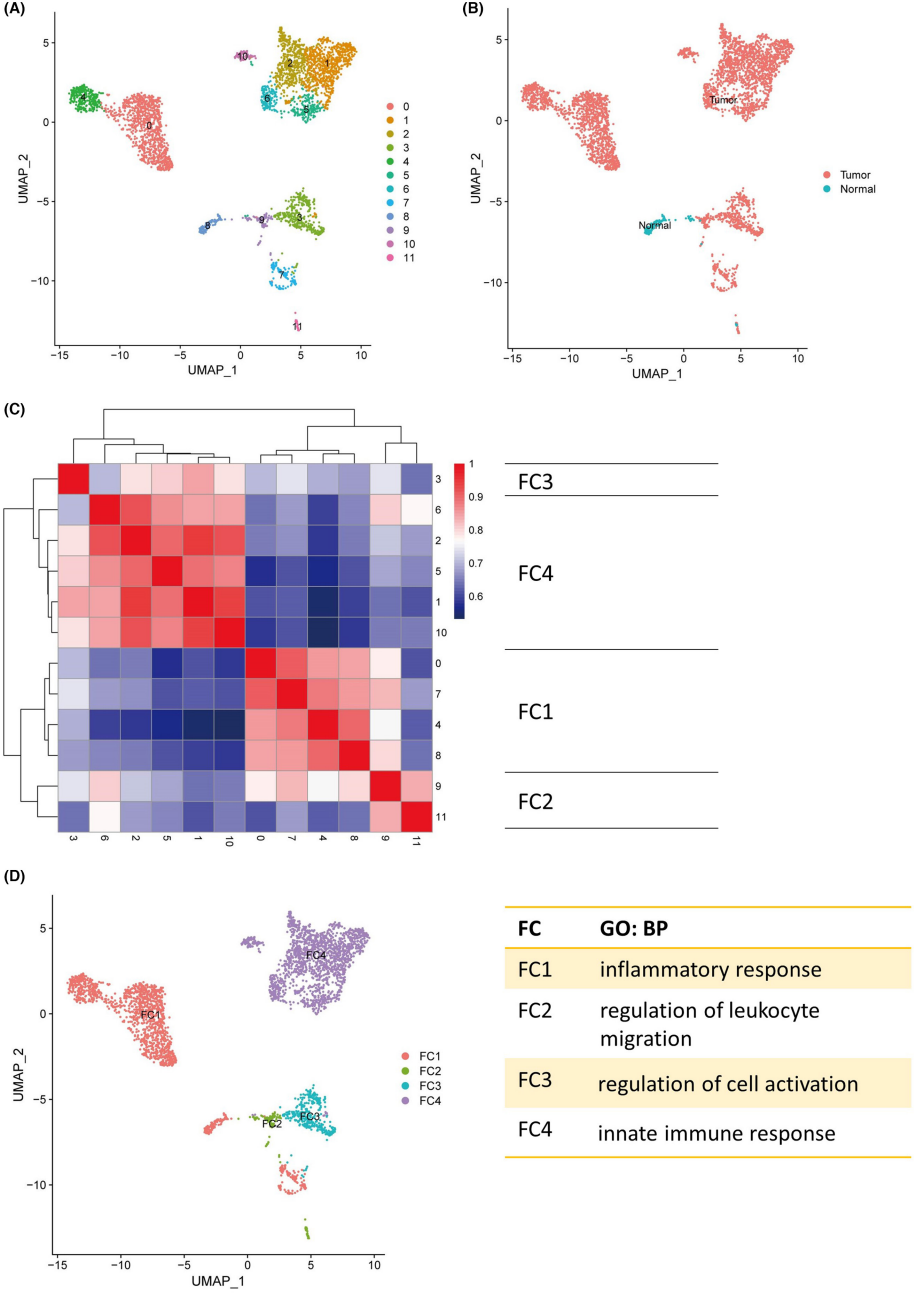
The comprehensive analysis of macrophages of ccRCC at single‐cell level resolution. (A) The Seurat clusters of macrophages. (B) The group clusters of macrophages. (C) The markable gene average expression heatmap of Seurat clusters of macrophages, and the red square represents high level of Pearson correlation coefficient, while blue square represents low level of Pearson correlation coefficient. (D) The FC clusters of macrophages, and the GOBP enrichment of FC clusters.

For the analysis of tumour‐associated macrophages (TAMs) in KIRC, macrophages were isolated from 252 normal kidney tissue samples and 1164 tumour tissue samples as depicted in Figure 2B. UMAP analysis illustrated macrophage heterogeneity, resulting in 12 identifiable clusters (Figure 2A). The gene expression profiles of these clusters diverged significantly, allowing for the classification of macrophages into four distinct functional subtypes (Figure 2C). Gene Ontology Biological Process (GOBP) annotations for subtype markers indicated that FC1 macrophages predominantly mediate immune responses, FC2 govern leukocyte migration, FC3 regulate cell activation and FC4 are involved in the innate immune response (Figure 2D).

### 3.3 Pseudotime analysis of macrophages of KIRC

**FIGURE 3:**
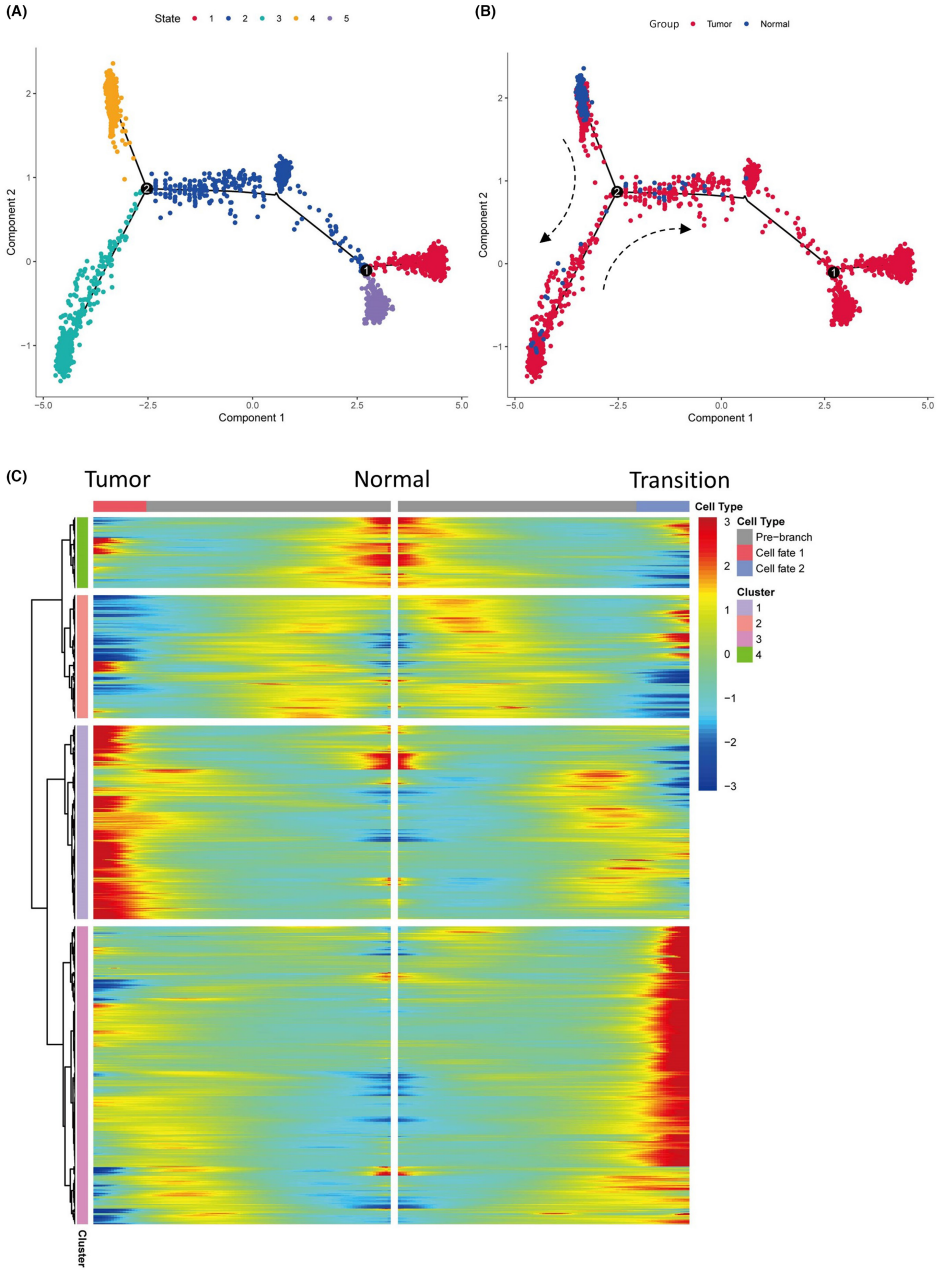
The pseudotime analysis of macrophages of ccRCC. (A) The trajectory map of macrophages of ccRCC in state types. (B) The trajectory map of macrophages of ccRCC in group types. (C) The BEAM heatmap of macrophages, and the red point represents high expression of gene while blue point represents low expression of gene.

The macrophage population of 1416 cells were mapped onto a genetic trajectory distribution, which highlighted a marked transitioning from a normal to a pathogenic cancer‐associated state (Figure 3A, B). Macrophages originating from healthy renal tissue predominantly constituted the left side of the trajectory, while those from cancerous tissue were more prevalent on the right, and the bottom portion contained macrophages from both sources. Differential gene expression analysis across normal, transitional and tumour‐associated macrophage states revealed distinctive profiles: a robust immune activation characterized the cancer‐associated state, whereas normal macrophages showed heightened metabolic pathway activity, and transitional macrophages exhibited an upregulation of glucose metabolism‐related pathways (Figure 3C). Genes enriched in cluster 1 were subsequently analysed to determine their prognostic significance.

### 3.4 CellChat analysis of KIRC

**FIGURE 4:**
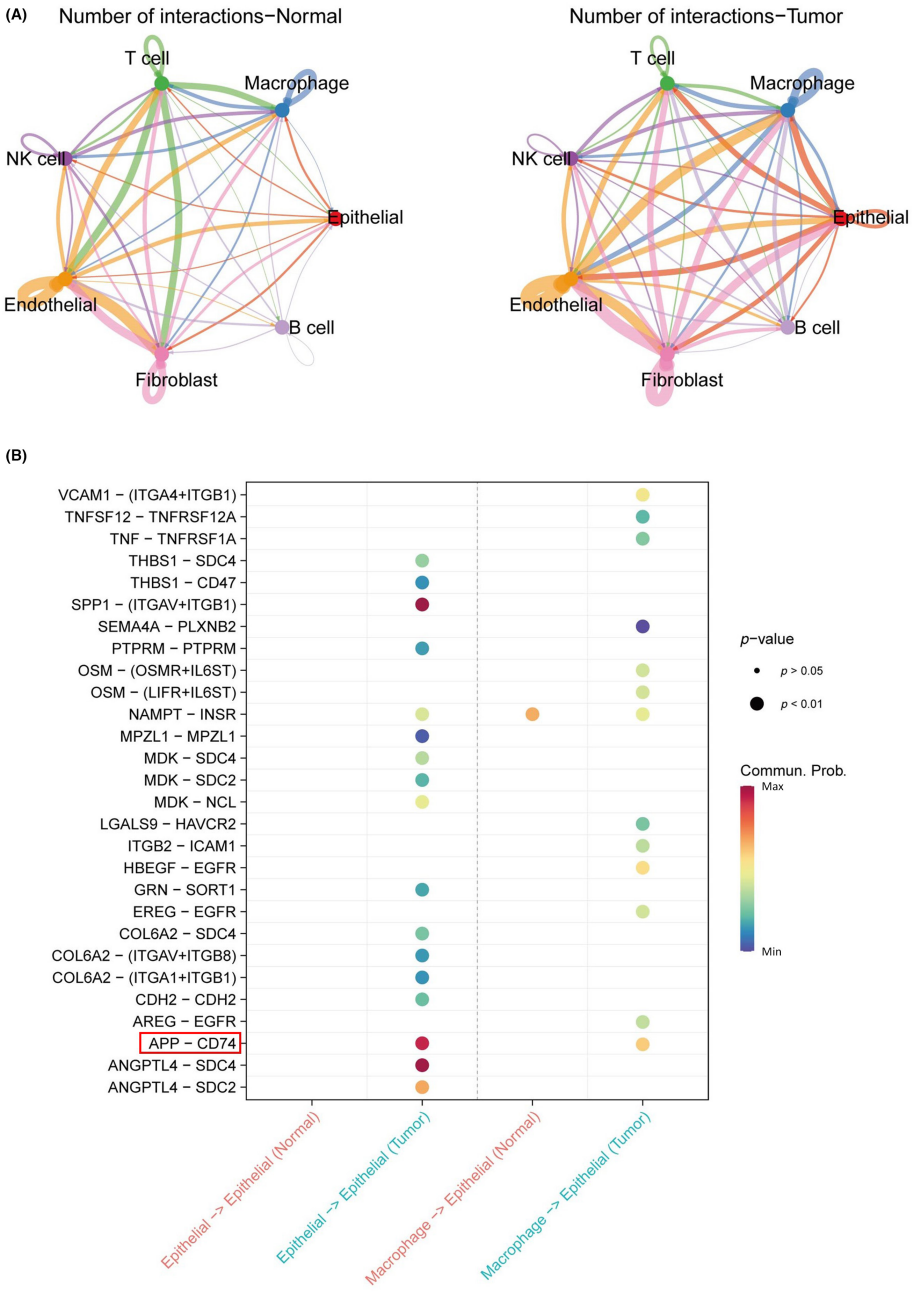
The CellChat analysis of ccRCC. (A) The network of cell–cell interactions. (B) The ligand–receptor pair of epithelial and macrophage.

Analysis of cellular interactions indicated an elevated intensity of cell‐to‐cell communication within the tumour tissue (Figure 4A), suggesting the profound impact of the rich immune microenvironment inherent to the malignant mass. In the receptor–ligand pair analysis, specific pairs were exclusively expressed in the tumour tissue (Figure 4B). Notably, the APP‐CD74 pair was involved in both tumour cell autocrine signalling and macrophage paracrine secretion, exhibiting intensified cellular communication.

### 3.5 Construction of TAM‐related genes prognostic model in KIRC

Utilizing the BEAM function, we identified a total of 299 genes in cluster 1. Subsequently, univariate Cox proportional hazards analyses yielded 123 prognostic genes from this set (*p* < 0.01), categorized into 61 high‐risk genes (HR > 1) and 62 low‐risk genes (HR < 1). Further, multivariate Cox proportional hazards analyses distilled these to 40 survival‐associated genes (*p* < 0.01), listed in Table S1. These genes formed the basis of a TAM‐related prognostic model. Stratification of KIRC patients into high‐ and low‐expression groups based on the median expression of these 40 genes revealed a statistically significant divergence in overall survival (OS) (*p* < 0.05) (Figure S1).

**FIGURE 5:**
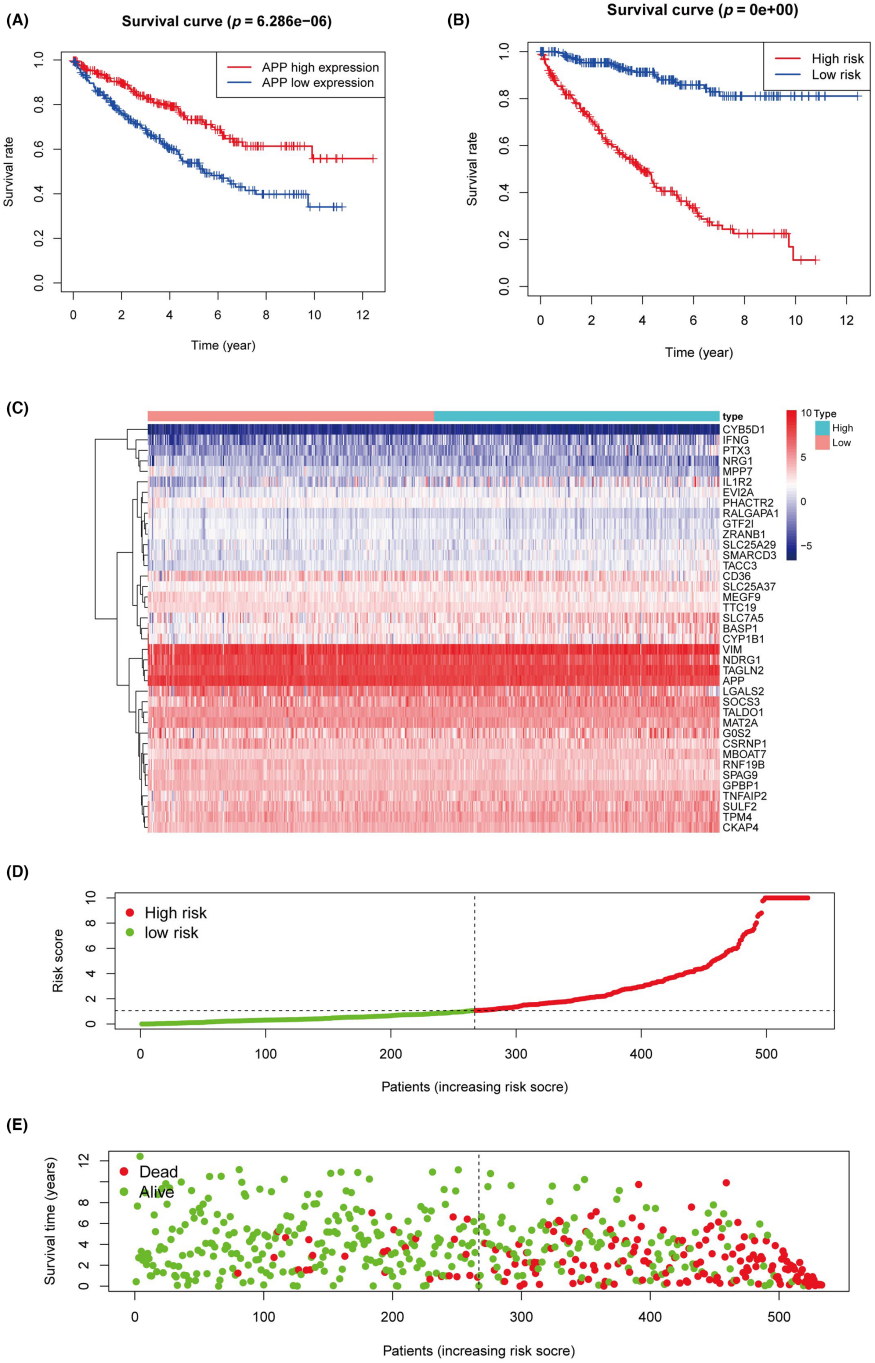
The construction of risk score. (A) The survival curve of APP. (B) The survival curve of risk score. (C) The heatmap displayed the expression levels of TAM‐related genes in the high‐risk and low‐risk groups. (D) The risk curve based on the risk score of each sample. (E) The scatterplot based on the survival status of each sample, and the green and red dots represent survival and death.

Through analysis of cellular communication, APP emerged as a gene of research interest due to its significant association with OS (*p* < 0.01) (Figure 5A). KIRC patients were divided into high‐risk and low‐risk groups according to the risk score median, with OS differences that were statistically significant (*p* < 0.01) (Figure 5B). The association between risk scores and mortality occurrence was depicted through both a risk curve and scatterplot (Figure 5D, E), while a heatmap conveyed the distinct expression profiles of the 40 TAM‐related genes in KIRC patients (Figure 5C).

**FIGURE 6:**
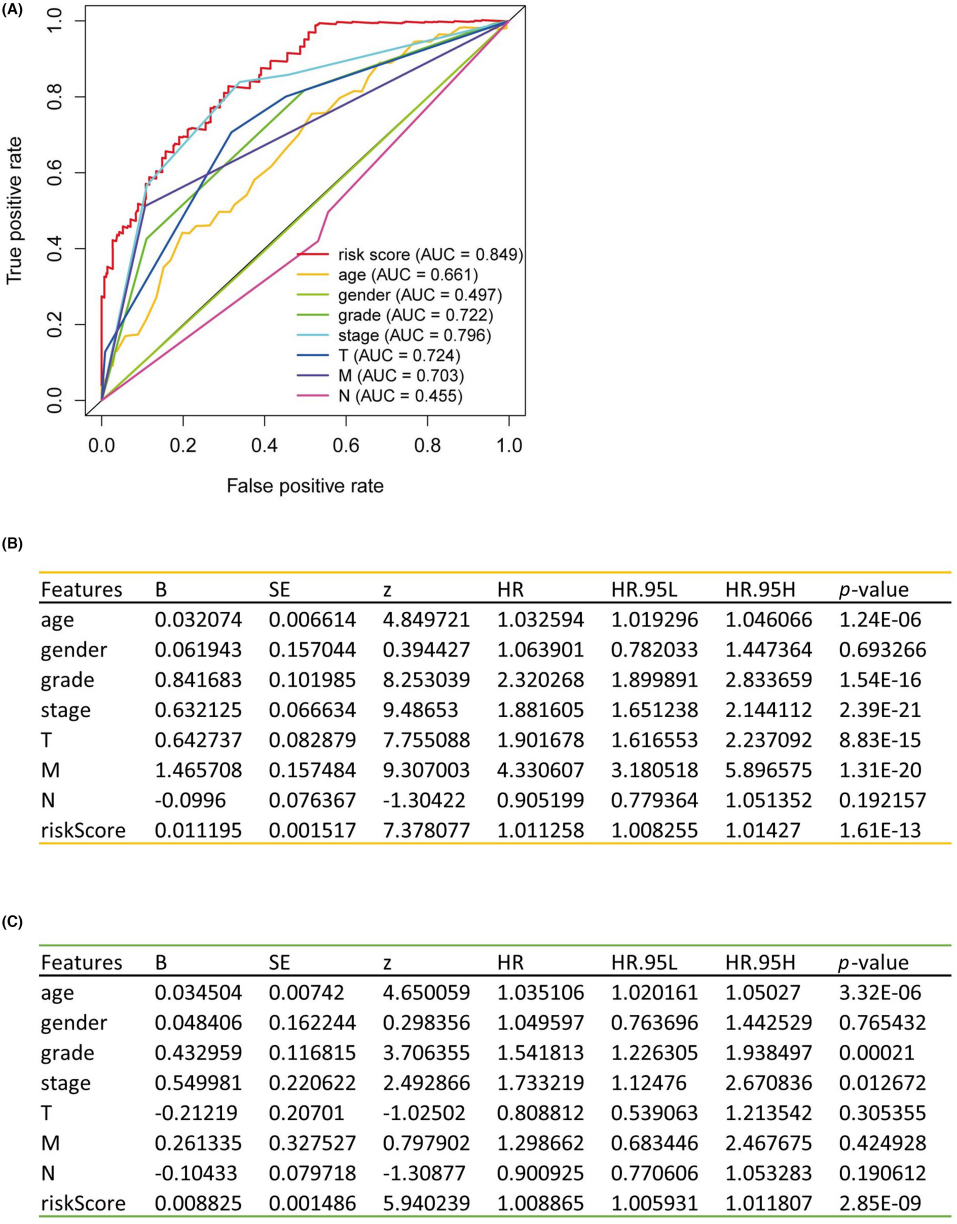
The comprehensive validation of risk score. (A) The ROC curve of risk score. (B) The results of univariate Cox regression analysis of risk score and other clinical factors. (C) The results of multivariate Cox regression analysis of risk score and other clinical factors. Clinical factors: age, gender, stage, T (tumour size), N (lymph node metastasis) and M (distant metastasis).

To determine the independence of the risk score as a prognostic factor for patients with KIRC, both univariate and multivariate Cox regression analyses were executed. The hazard ratio (HR) for the risk score was 1.011 (95% CI 1.008–1.014) (*p* < 0.01) from the univariate analysis (Figure 6B) and 1.008 (95% CI 1.005–1.011) (*p* < 0.01) from the multivariate analysis (Figure 6C). Consequently, the risk score qualifies as an independent prognostic indicator for patients with KIRC. Notably, the area under the curve (AUC) for the risk score was 0.849, outperforming other clinical factors and highlighting its prognostic prediction potential in KIRC patients (Figure 6A).

### 3.6 Cross validation of APP

**FIGURE 7:**
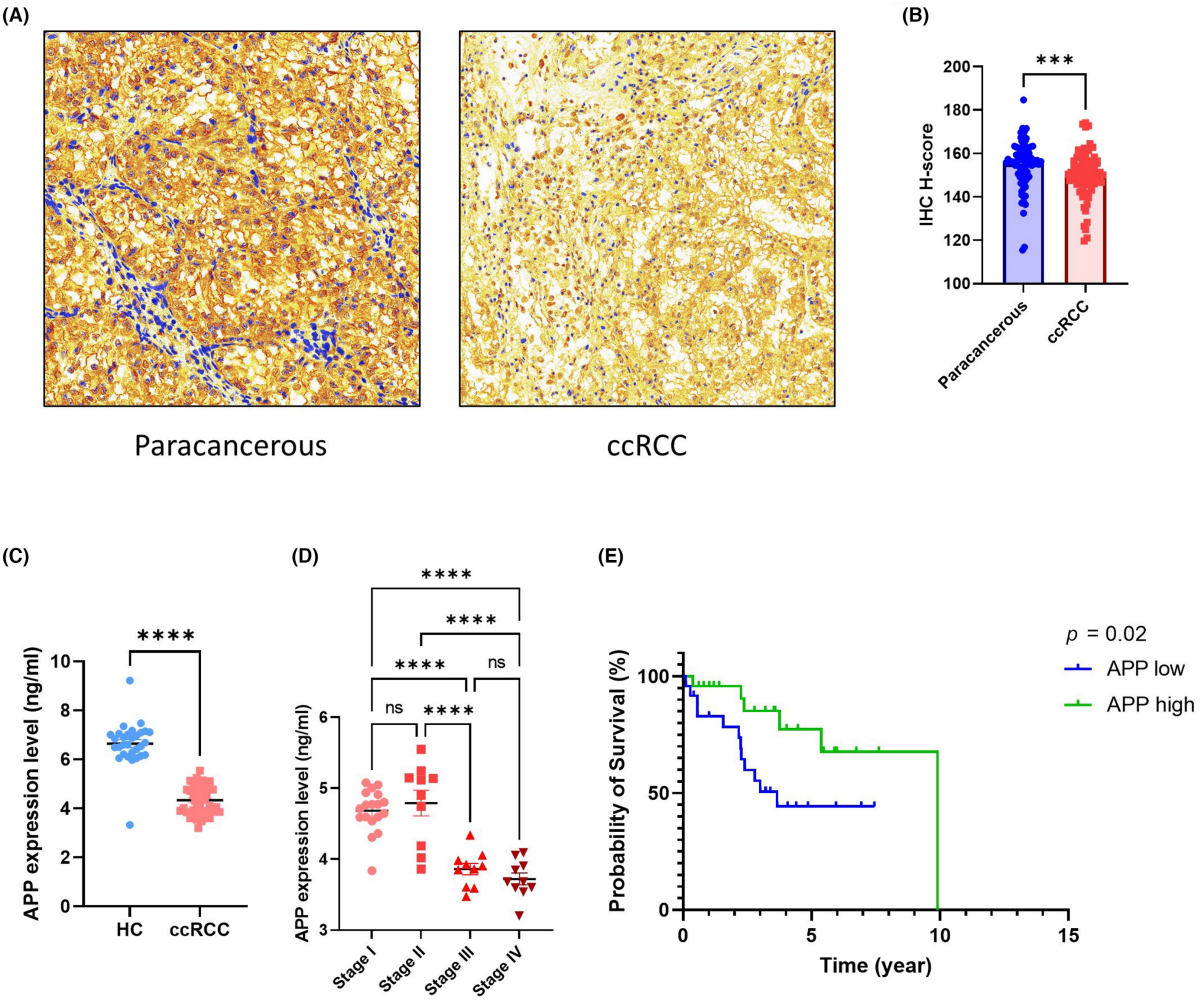
The expression level of APP. (A) The IHC representative images of APP. (B) The statistical results of IHC. (C) The serum expression level of APP in HC and ccRCC groups. (D) The serum expression level of APP in different stage ccRCC groups. (E) The survival curve of APP low group and APP high group.

**FIGURE 8:**
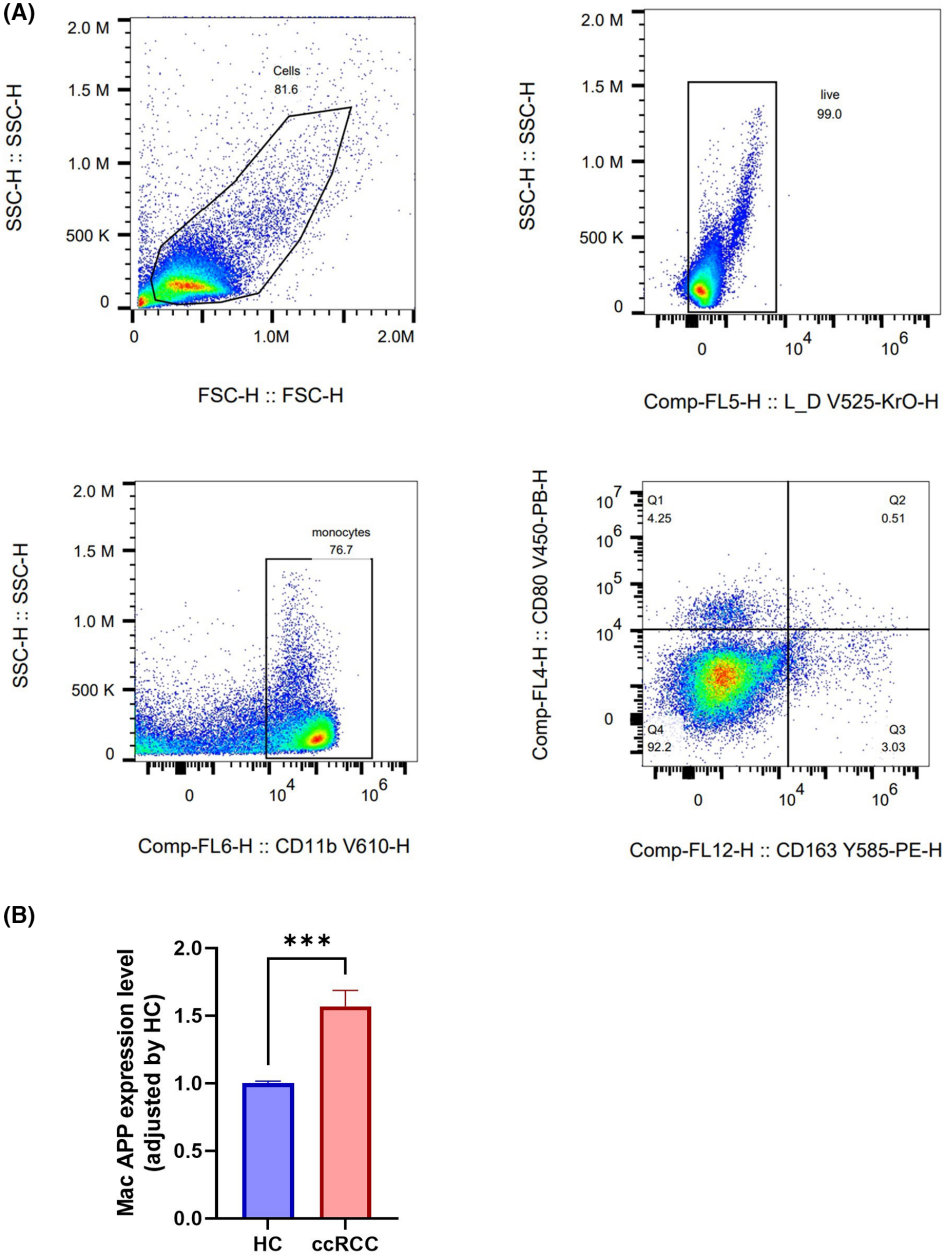
The expression level of APP in macrophage. (A) The flow sorting strategic of macrophage. (B) The expression level of APP in macrophage.

Upon investigating 40 TAM‐related genes, we identified their prognostic relevance as evidenced by Kaplan–Meier (KM) survival plots. Of these, only APP exhibited significant prognostic value coupled with a pivotal role in cellular communication. Tissue microarray analyses revealed that APP expression was elevated in peritumoral compared to tumour tissues (Figure 7A, B). ELISA assays on plasma samples from individuals with KIRC and healthy controls indicated higher APP levels in the latter (Figure 7C). Additionally, when comparing stages of KIRC, ELISA results demonstrated that APP was most abundant in stages I and II and diminished in stages III and IV of the disease (Figure 7D). Patients were categorized into APP‐high and APP‐low groups according to their plasma APP concentrations in KIRC, and the resultant survival curves indicated a more favourable prognosis for those with elevated APP levels (Figure 7E). In primary macrophages isolated from KIRC, increased APP secretion was observed (Figure 8A, B). Among the primary macrophages, the ones isolated from tumour was higher proportion of immune cells than the ones isolated from renal malformation [28.82% (95% CI: 23.45%–33.49%) vs. 12.85% (only one renal malformation sample)].

## 4 DISCUSSION

Renal cell carcinoma (RCC), particularly clear cell renal cell carcinoma (ccRCC), is a significant challenge in oncology. Our study's focus on the tumour microenvironment (TME) of ccRCC, especially the role of APP in tumour‐associated macrophages (TAMs), offers novel insights into the disease's progression.^14^, ^18^ We discovered that APP expression in TAMs correlates with patient prognosis, suggesting its potential as a novel biomarker in ccRCC.^19^ This insight is crucial given the pivotal role of the TME in RCC progression and response to therapy.^20^, ^21^ We used single‐cell data screening to obtain TAM‐related genes and constructed a prognostic model based on TAM‐related genes. At present, we are the first prognostic model constructed based on TAM‐related genes in the field of renal clear cell carcinoma.

Clinical research has shown that immune checkpoint inhibitors (ICIs) such as nivolumab and ipilimumab have become important treatment options for advanced RCC.^22^ Studies involving multiplexed immunohistochemistry to define the TME, including various immune cell subsets and macrophages, have highlighted the predictive potential of these components in treatment response.^23^, ^24^ In a variety of cancers, the TME has become the focus of current research. In addition, it plays a crucial role in cancer treatment and prevention. The RCC TME is highly dynamic, adaptive and heterogeneous, posing challenges in treatment resistance. Anti‐angiogenic therapies and immunotherapies have revolutionized RCC treatment, but their effectiveness can be hampered by the TME's complexity.^25^ Understanding the components of the RCC TME and their contribution to disease progression is essential for improving therapeutic strategies.

Recent research has shed light on the diverse roles of APP in cancer, particularly in signalling pathways. For instance, in breast cancer, APP has been shown to promote cell migration and invasion by activating the mitogen‐activated protein kinase (MAPK) signalling pathway, which is crucial for epithelial‐mesenchymal transition (EMT) and metastasis.^15^, ^26^ This finding highlights the potential of APP in influencing key cancer processes through signalling pathways, suggesting a similar mechanism could be at play in ccRCC.^16^, ^17^ Clinical trials targeting the TME in RCC, including the role of TAMs and specific molecules like APP, are essential to advance our understanding and management of ccRCC.^27^ According to the results of TCGA, APP is a protective secreted protein, and groups with high APP expression levels have longer survival and better prognosis. However, according to the single‐cell data, the high levels of APP expression in macrophages in the tumour state are indicated. This is consistent with the results of our flow sorting. We believe that APP is secreted by macrophages and plays a protective role, which is lost when APP is depleted, leading to tumour progression.

Our predictive model, demonstrating an AUC of 0.849, underscores the importance of molecular and cellular heterogeneity within the ccRCC microenvironment. This model challenges the traditional staging system by providing a more nuanced understanding of ccRCC progression, highlighting the critical role of the TME and signalling pathways in cancer development.

However, our study has limitations. Further research is needed to elucidate the specific mechanisms of APP in ccRCC and validate our predictive model in a larger cohort. Additionally, exploring the signalling pathways influenced by APP in ccRCC could provide deeper insights into the disease's pathogenesis and potential therapeutic targets. Besides, the specific regulatory mechanisms of APP in ccRCC and its direct clinical implications require further exploration. The development of therapies targeting APP within the TME of ccRCC is an area ripe for future clinical research. In addition, APP‐specific protection mechanisms need to be explored further experimentally.

In future research, we aim to explore the regulatory mechanisms of APP in the TME of ccRCC, especially its role in signalling pathways like MAPK, which could offer new avenues for therapeutic intervention. Understanding APP's influence on cancer cell behaviour and interaction with the TME could revolutionize ccRCC treatment strategies.

## 5 CONCLUSION

In this study, we employed the scRNA‐seq analysis and transcriptomics method to successfully identify distinct TAM subtypes of TME cells. Furthermore, we elucidated the role of APP in regulating of tumour protection and prevention of tumour progression, serving as important prognostic markers and indicators in ccRCC cohorts. Finally, we developed a prognostic model based on TAM‐related genes with better efficacy in assessing prognosis.

## AUTHOR CONTRIBUTIONS

**Guan Bo:** Formal analysis (lead); writing – original draft (equal). **Ming Li:** Methodology (lead). **Di Cui:** Writing – original draft (equal). **Shen Chen:** Writing – review and editing (lead). **Zongyao Hao:** Supervision (equal). **Xiaowei Li:** Supervision (equal).

## FUNDING INFORMATION

Scientific research project of Fuyang Health Commission of Anhui Province in 2021 (FY2021‐003). Clinical Medical Research Translation Special Foundation of Anhui Province (202204295107020031; 202204295107020053).

## CONFLICT OF INTEREST STATEMENT

The authors declare no conflict of interest.

## Supporting information


Figure S1.

Table S1.

## Data Availability

All data used in this study were acquired from The Cancer Genome Atlas (TCGA) portal and GEO database. Each patient participating in the study signed an informed consent.
