# Supplementary material for: Single‐cell transcriptomic analysis in clear cell renal cell carcinoma: Deciphering the role of APP within the tumour microenvironment
Source: J Cell Mol Med. 2024 Mar 6;28(6):e18186. doi: 10.1111/jcmm.18186 (PMC10915830; doi:10.1111/jcmm.18186)
Supplement: Supplementary file 1 — Figure S1. [file JCMM-28-e18186-s001.pdf]

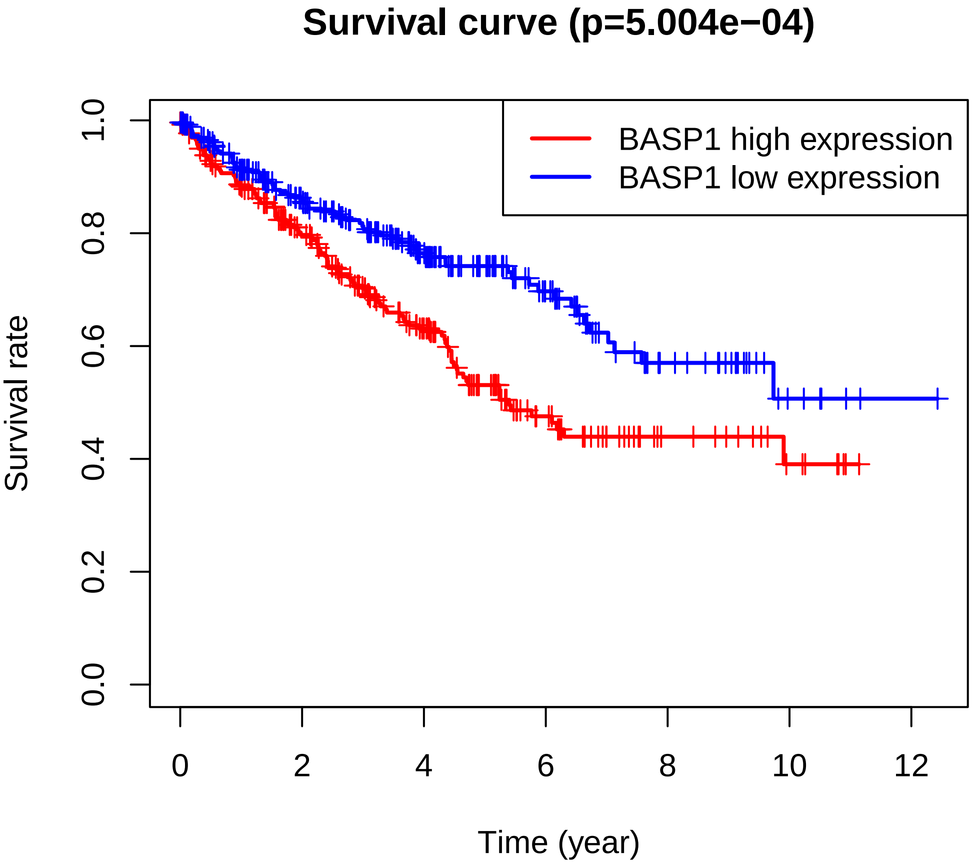
Survival curve (p=5.004e−04)
BASP1 high expression
BASP1 low expression
Survival rate
Time (year)
0.0
0.2
0.4
0.6
0.8
1.0
0
2
4
6
8
10
12

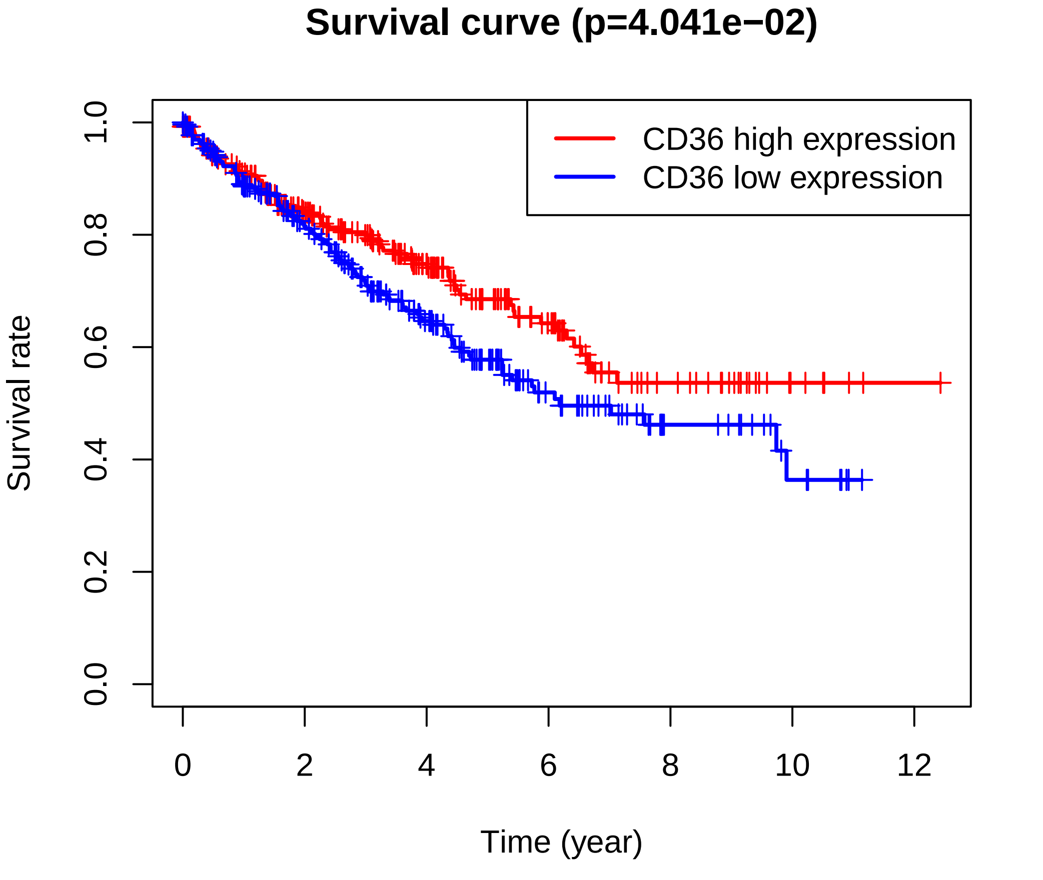
Survival curve (p=4.041e−02)
CD36 high expression
CD36 low expression
Survival rate
Time (year)
0.0
0.2
0.4
0.6
0.8
1.0
0
2
4
6
8
10
12

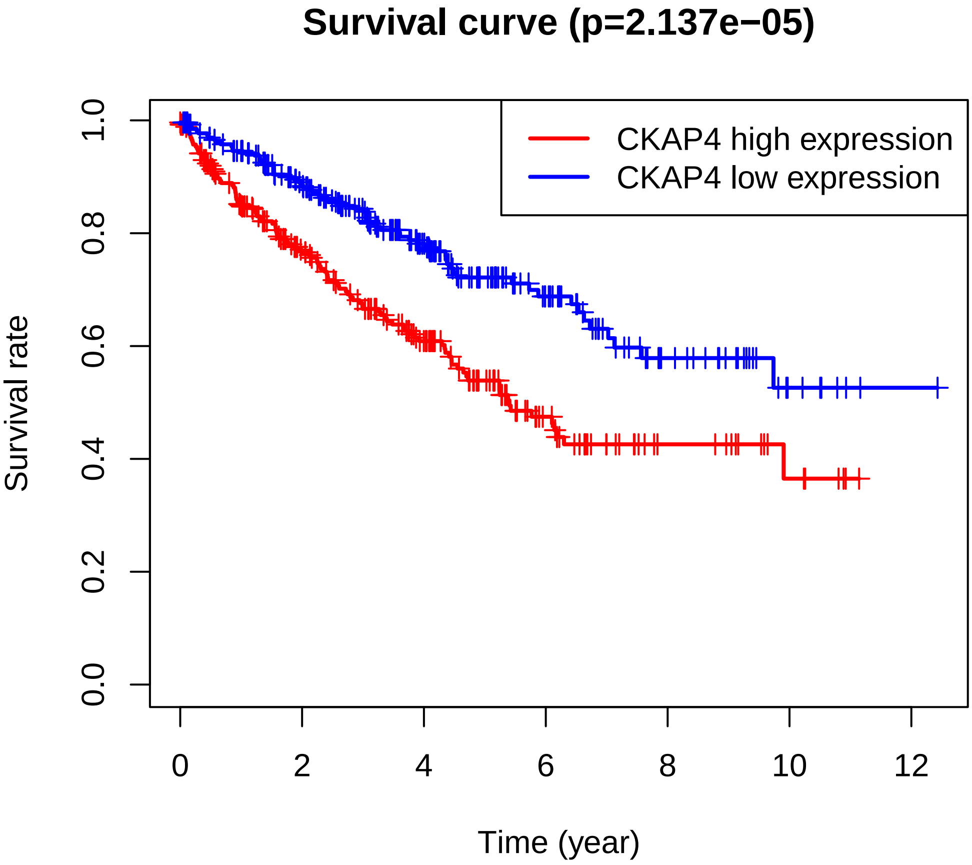
Survival curve (p=2.137e−05)
CKAP4 high expression
CKAP4 low expression
Survival rate
0.0
0.2
0.4
0.6
0.8
1.0
0
2
4
6
8
10
12
Time (year)

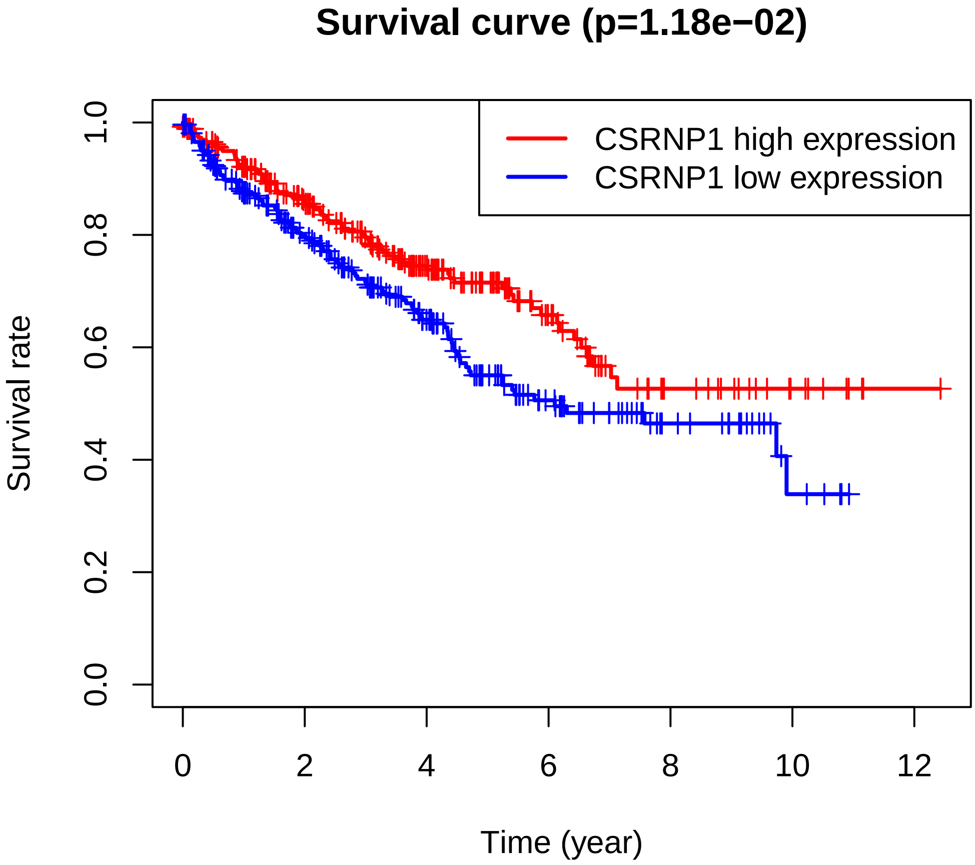
Survival curve (p=1.18e−02)
CSRNP1 high expression
CSRNP1 low expression
Survival rate
1.0
0.8
0.6
0.4
0.2
0.0
0
2
4
6
8
10
12
Time (year)

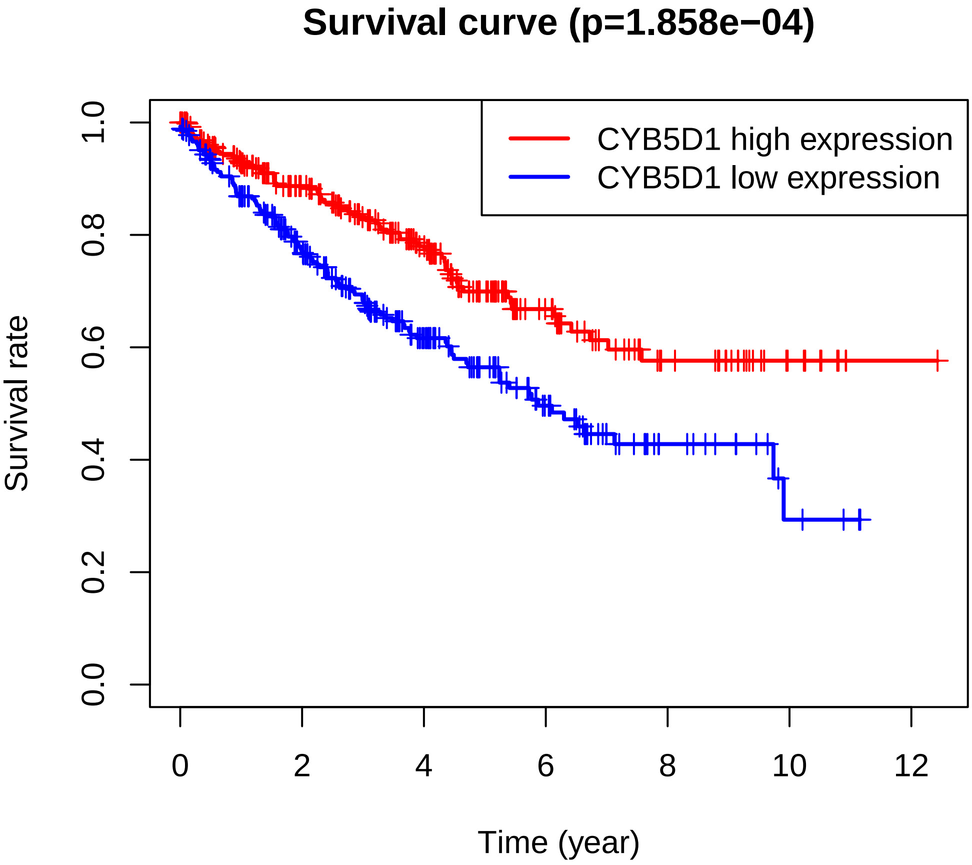
Survival curve (p=1.858e−04)
CYB5D1 high expression
CYB5D1 low expression
Survival rate
1.0
0.8
0.6
0.4
0.2
0.0
0
2
4
6
8
10
12
Time (year)

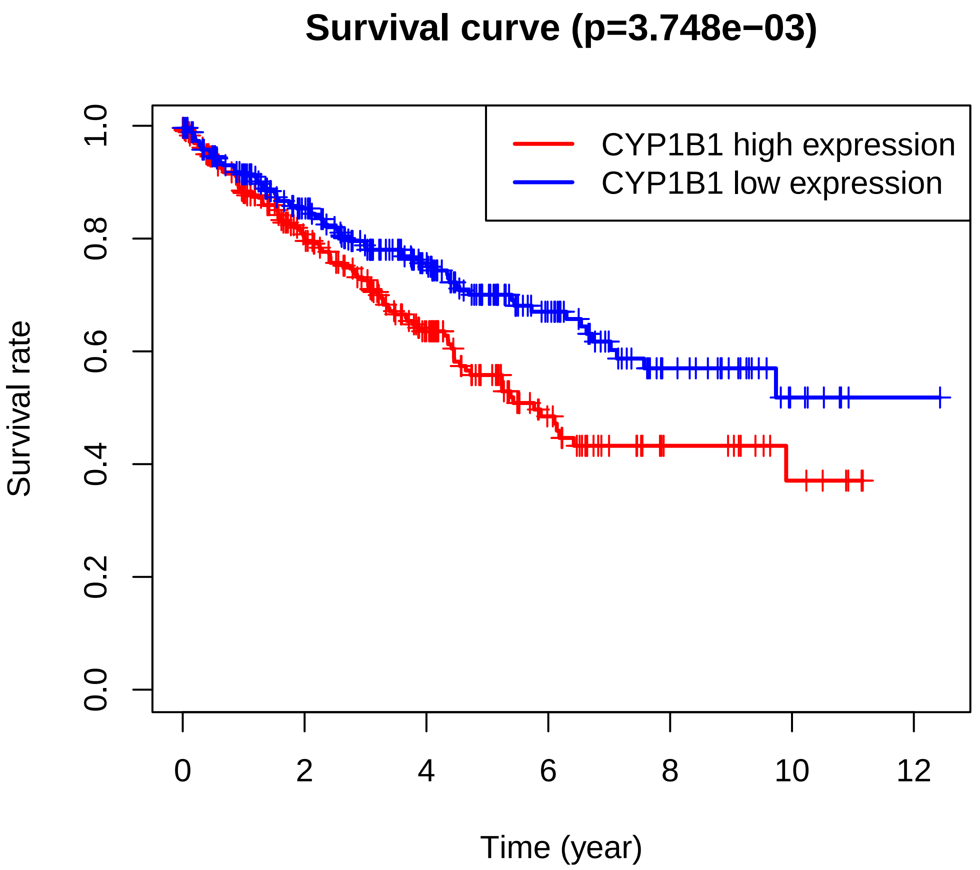
Survival curve (p=3.748e−03)
CYP1B1 high expression
CYP1B1 low expression
Survival rate
1.0
0.8
0.6
0.4
0.2
0.0
0
2
4
6
8
10
12
Time (year)

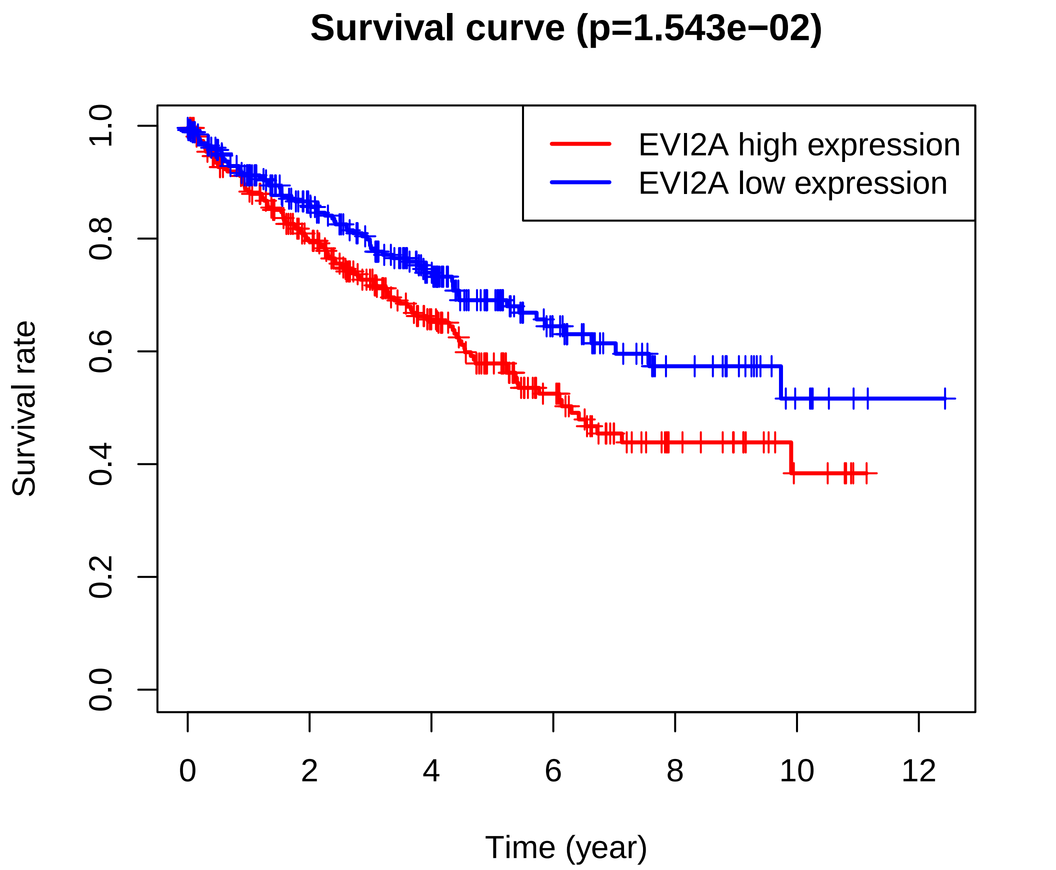

Survival curve (p=1.543e−02)
EVI2A high expression
EVI2A low expression
Survival rate
1.0
0.8
0.6
0.4
0.2
0.0
0
2
4
6
8
10
12
Time (year)

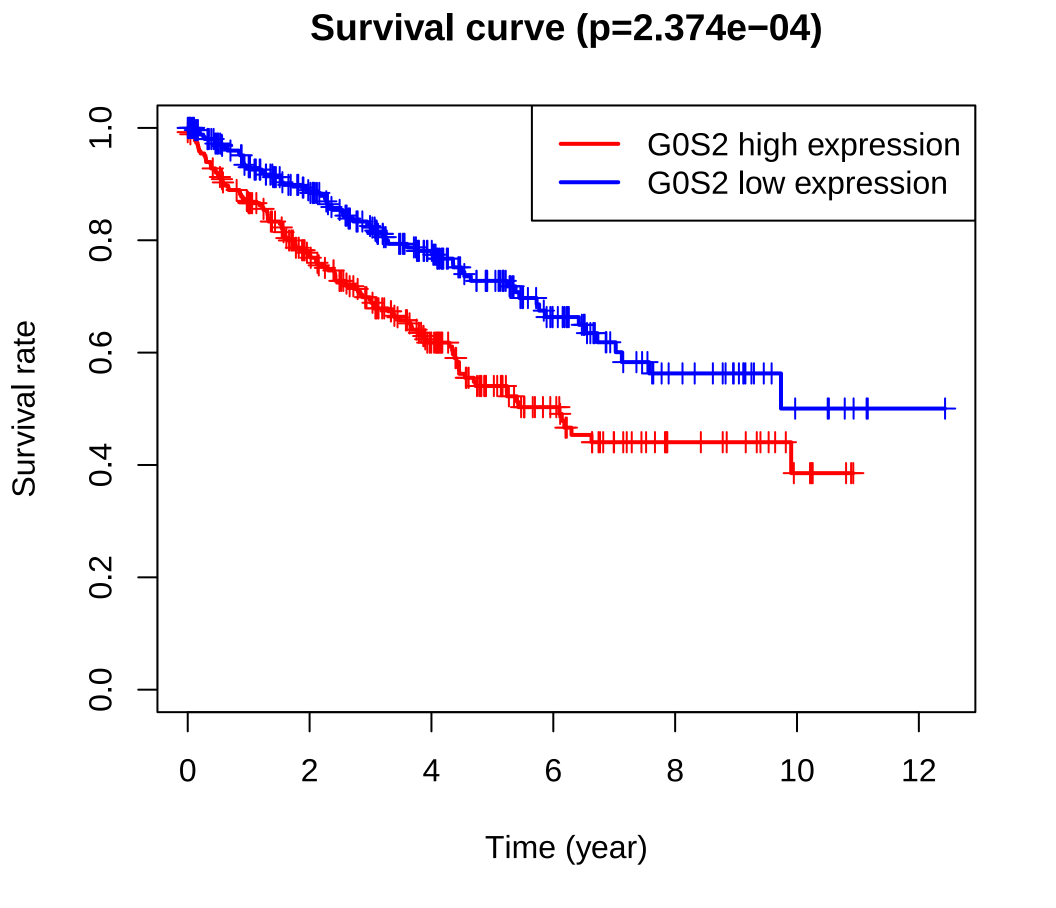

Survival curve (p=2.374e−04)
G0S2 high expression
G0S2 low expression
Survival rate
Time (year)
0.0
0.2
0.4
0.6
0.8
1.0
0
2
4
6
8
10
12

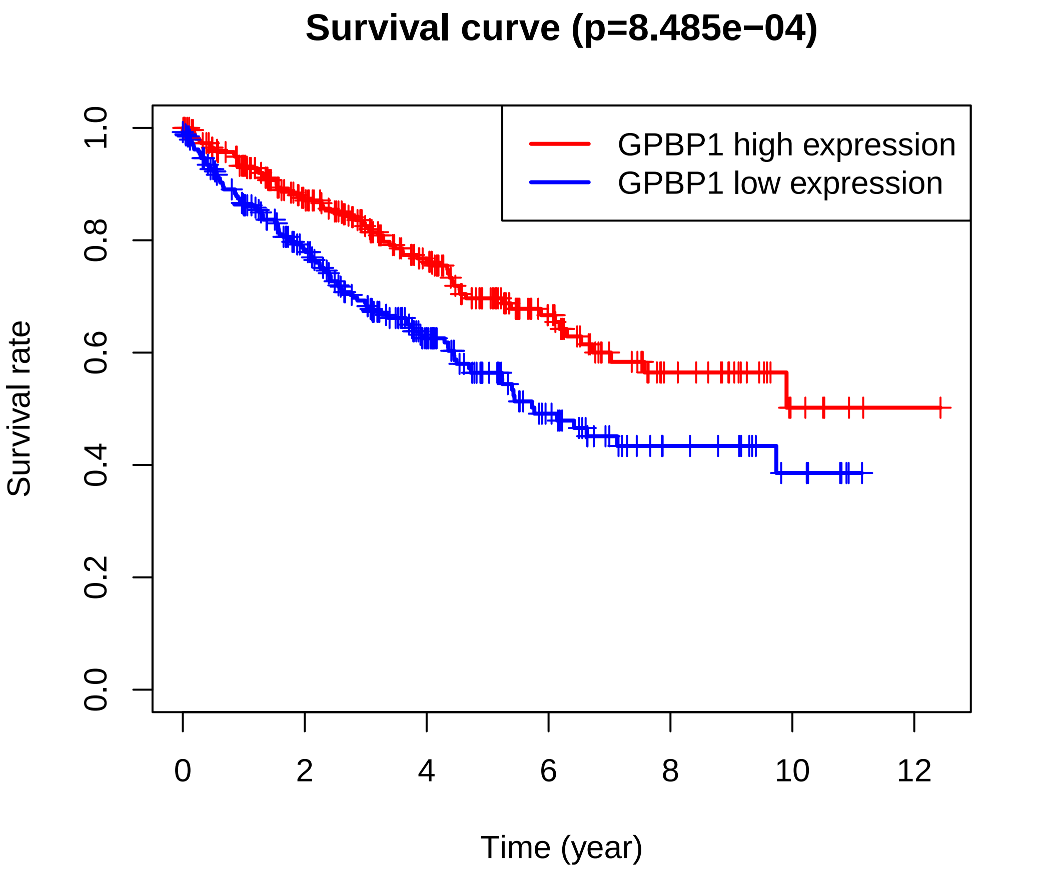
Survival curve (p=8.485e−04)
GPBP1 high expression
GPBP1 low expression
Survival rate
1.0
0.8
0.6
0.4
0.2
0.0
0
2
4
6
8
10
12
Time (year)

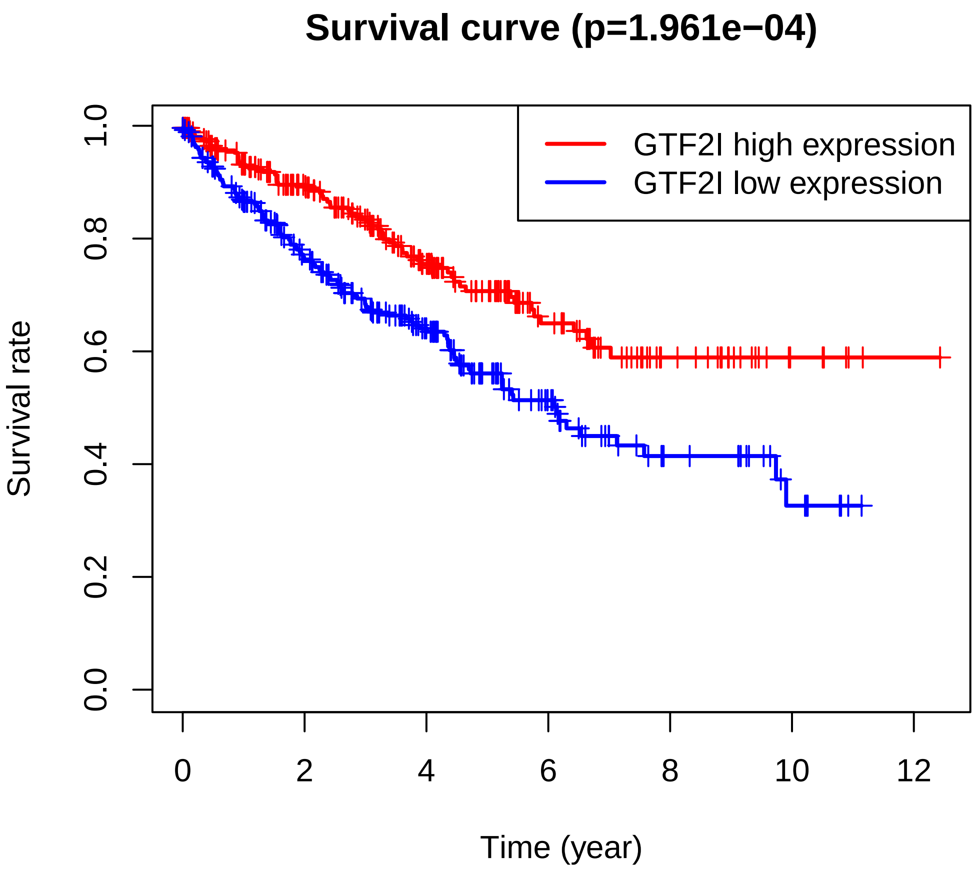
Survival curve (p=1.961e−04)
GTF2I high expression
GTF2I low expression
Survival rate
Time (year)
0.0
0.2
0.4
0.6
0.8
1.0
0
2
4
6
8
10
12

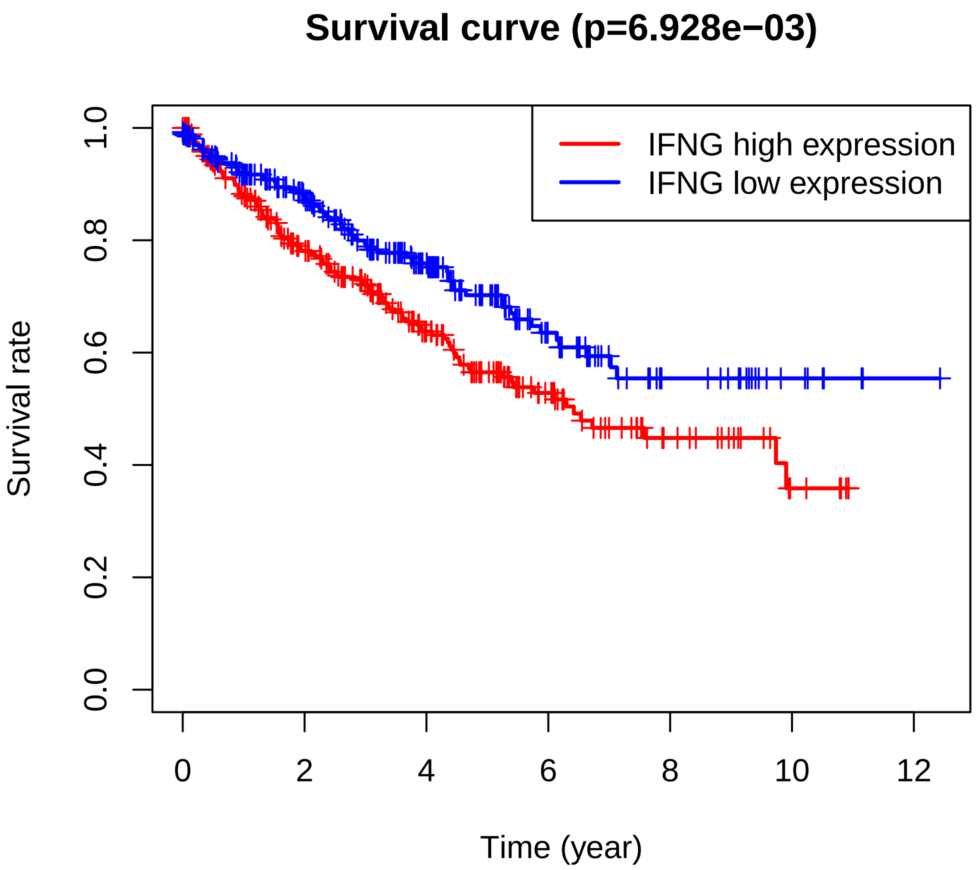
Survival curve (p=6.928e−03)
IFNG high expression
IFNG low expression
Survival rate
Time (year)
0.0
0.2
0.4
0.6
0.8
1.0
0
2
4
6
8
10
12

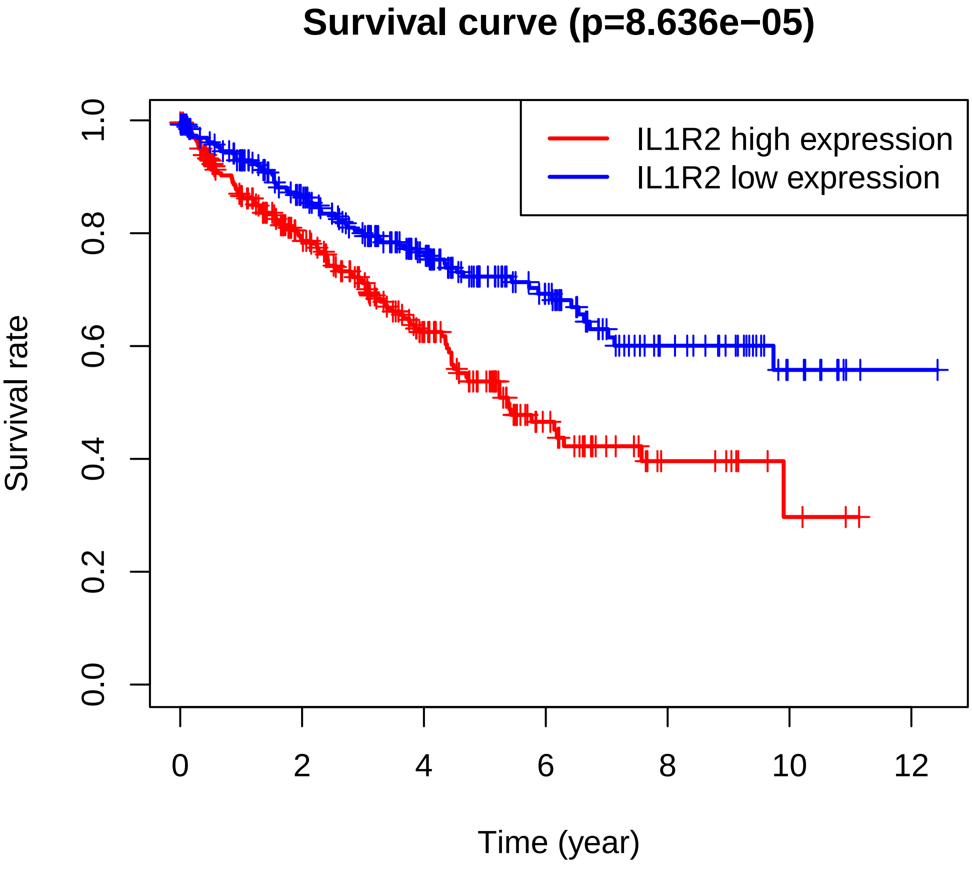
Survival curve (p=8.636e−05)
IL1R2 high expression
IL1R2 low expression
Survival rate
Time (year)
0.0
0.2
0.4
0.6
0.8
1.0
0
2
4
6
8
10
12

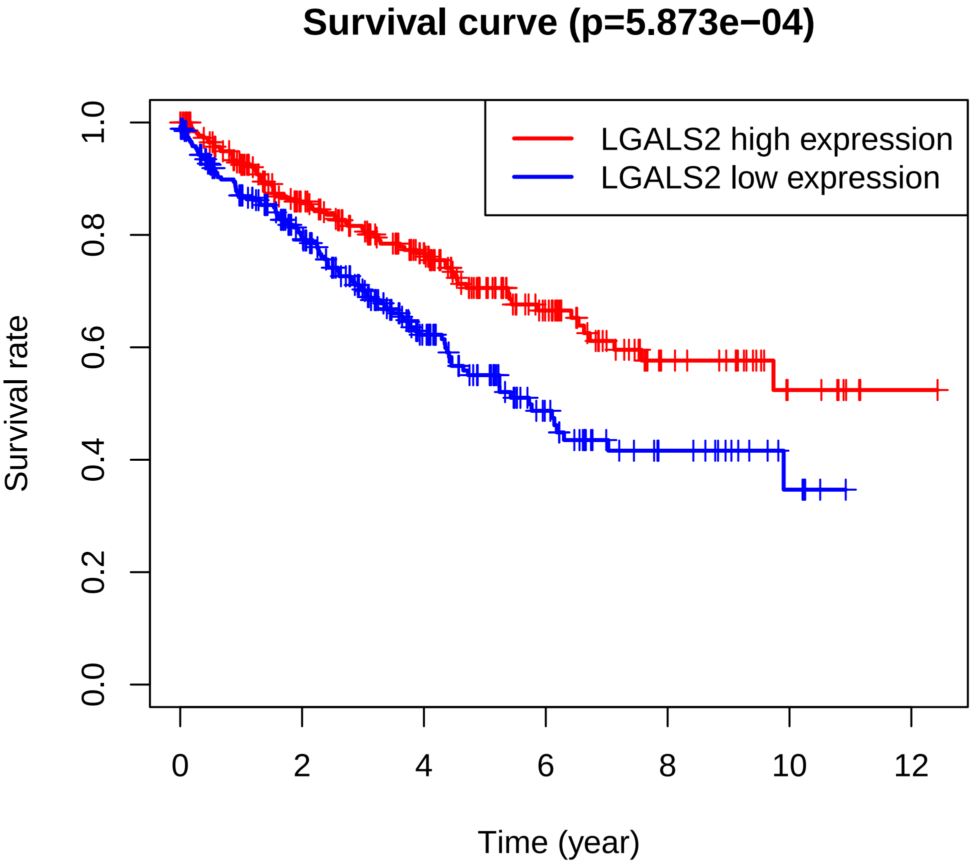

Survival curve (p=5.873e−04)
LGALS2 high expression
LGALS2 low expression
Survival rate
Time (year)
0.0
0.2
0.4
0.6
0.8
1.0
0
2
4
6
8
10
12

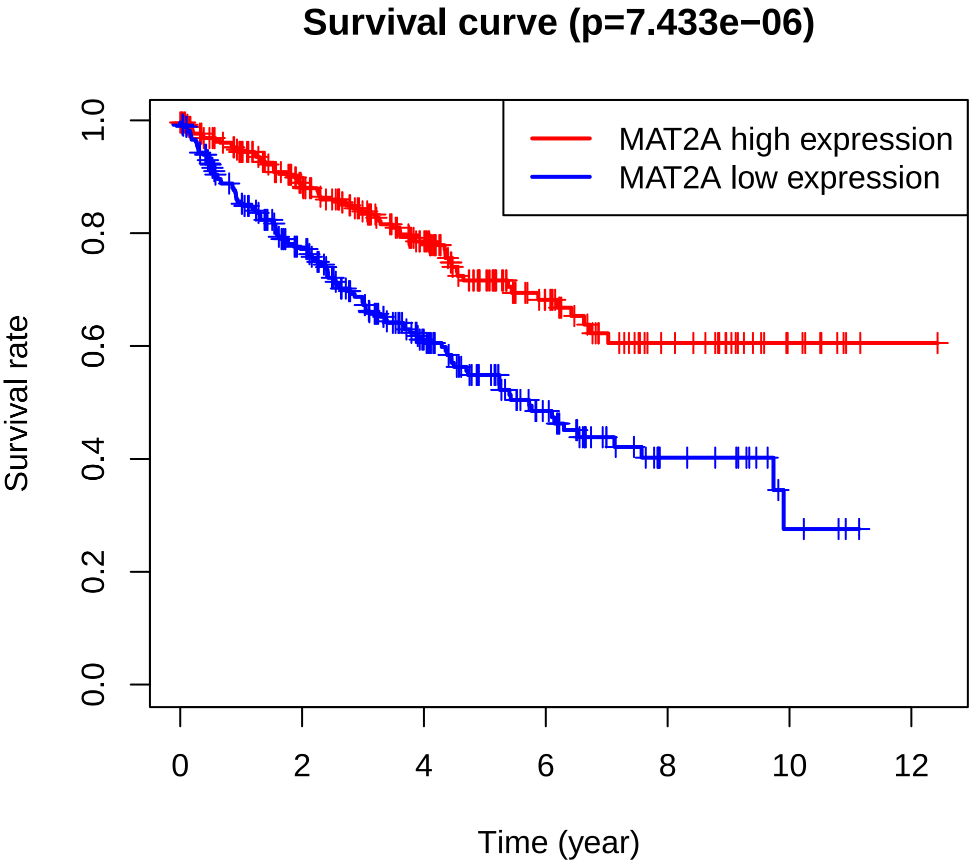
Survival curve (p=7.433e−06)
MAT2A high expression
MAT2A low expression
Survival rate
1.0
0.8
0.6
0.4
0.2
0.0
0
2
4
6
8
10
12
Time (year)

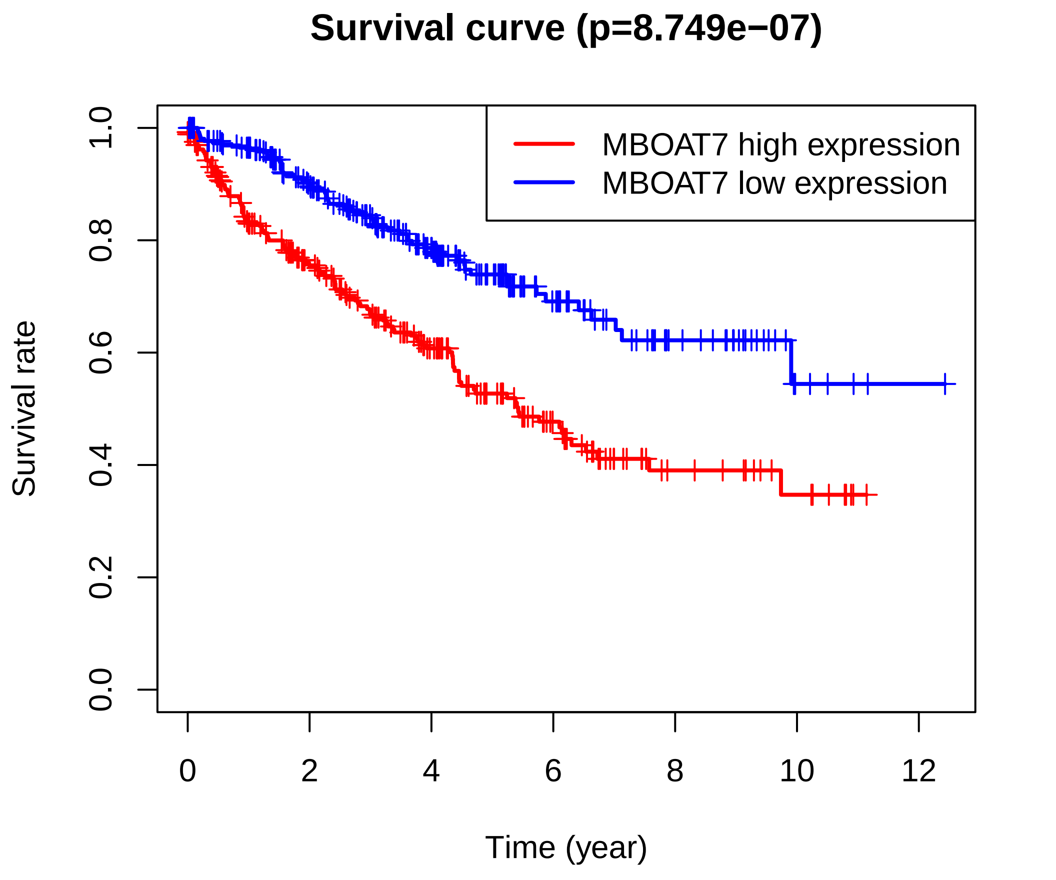

Survival curve (p=8.749e−07)
MBOAT7 high expression
MBOAT7 low expression
Survival rate
Time (year)
0.0
0.2
0.4
0.6
0.8
1.0
0
2
4
6
8
10
12

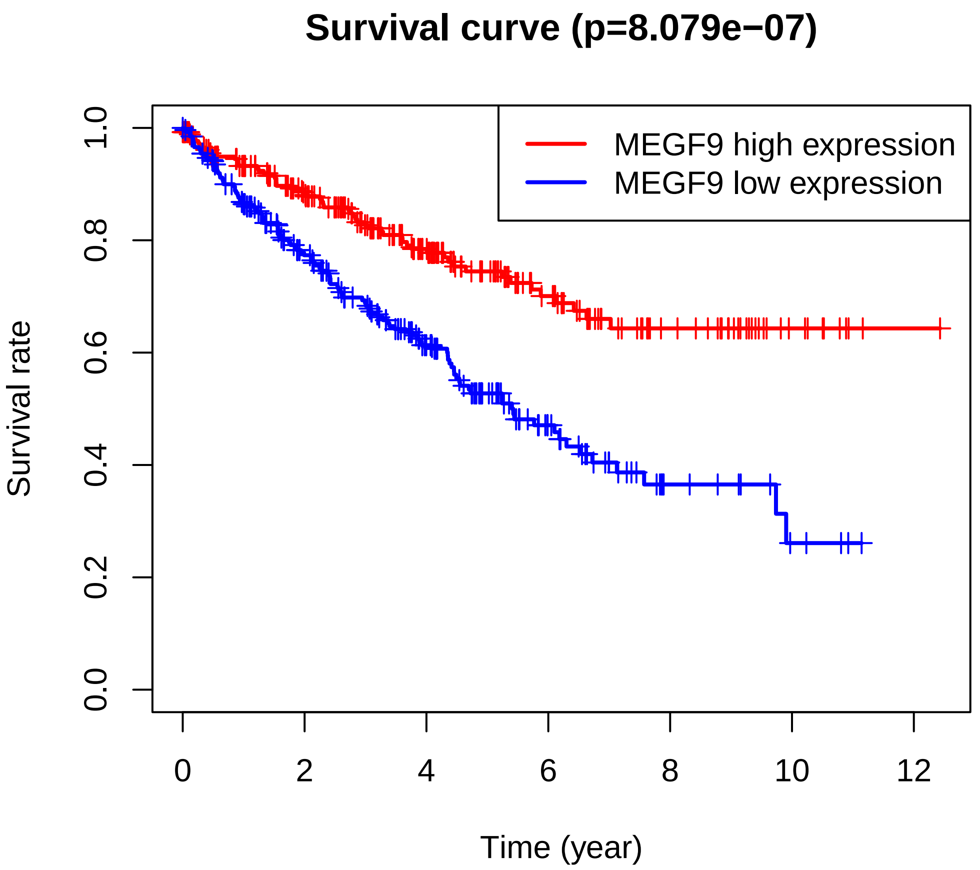

Survival curve (p=8.079e−07)
MEGF9 high expression
MEGF9 low expression
Survival rate
Time (year)
0.0
0.2
0.4
0.6
0.8
1.0
0
2
4
6
8
10
12

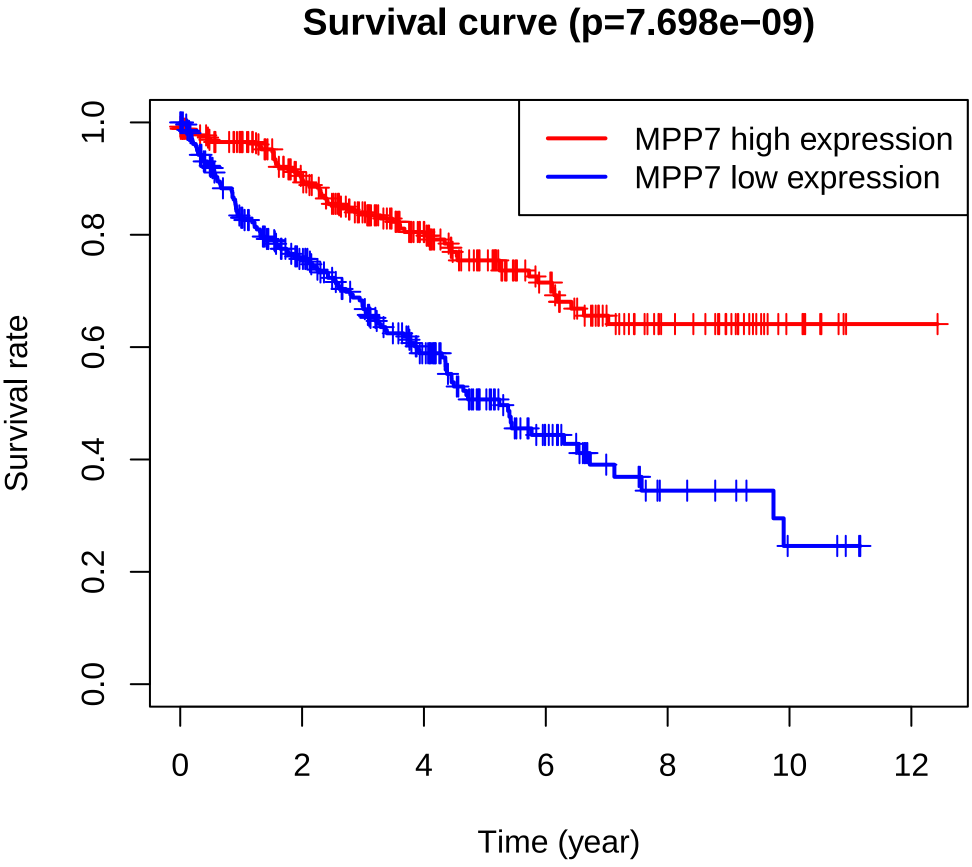

Survival curve (p=7.698e−09)
MPP7 high expression
MPP7 low expression
Survival rate
0.0
0.2
0.4
0.6
0.8
1.0
Time (year)
0
2
4
6
8
10
12

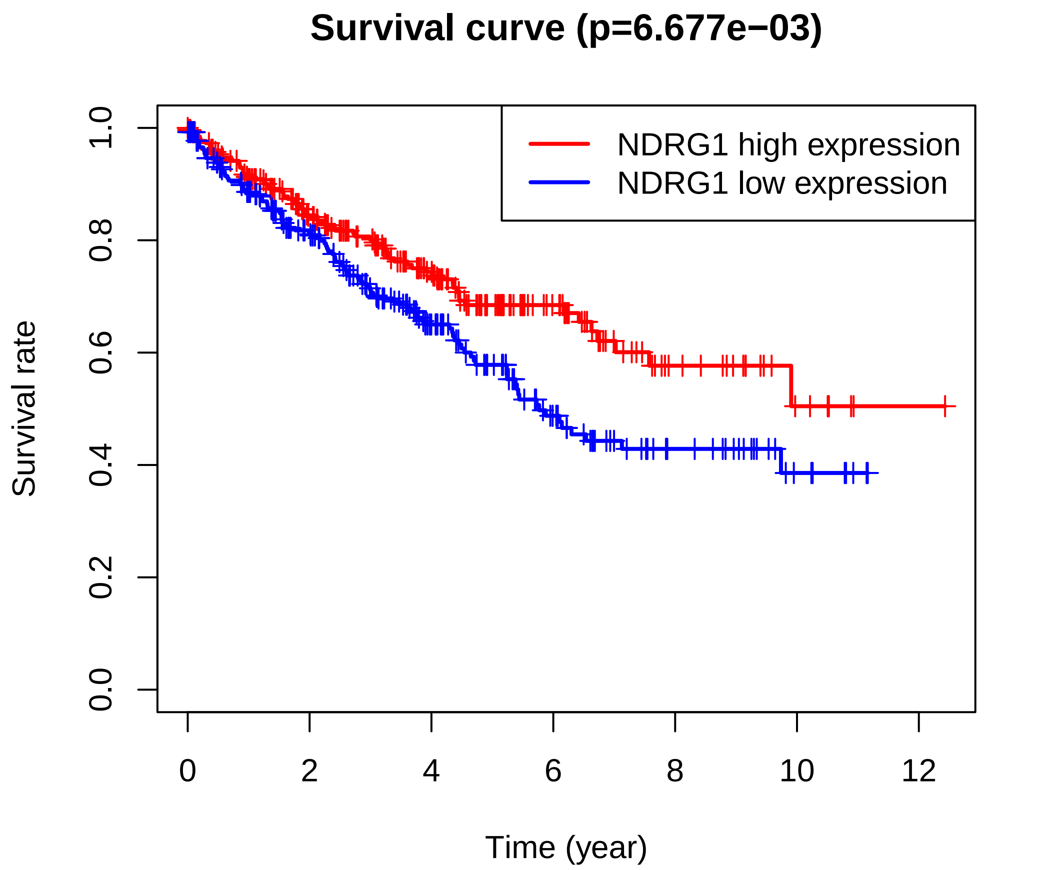
Survival curve (p=6.677e−03)
NDRG1 high expression
NDRG1 low expression
Survival rate
1.0
0.8
0.6
0.4
0.2
0.0
0
2
4
6
8
10
12
Time (year)

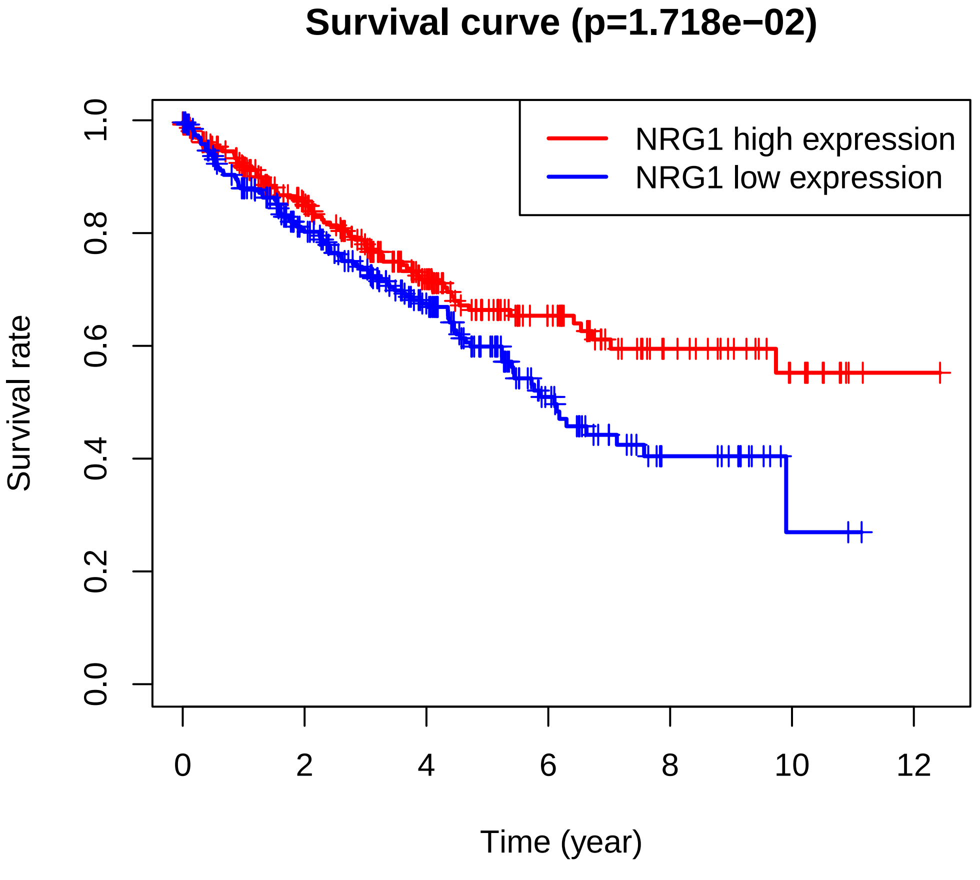
Survival curve (p=1.718e−02)
NRG1 high expression
NRG1 low expression
Survival rate
Time (year)
0.0
0.2
0.4
0.6
0.8
1.0
0
2
4
6
8
10
12

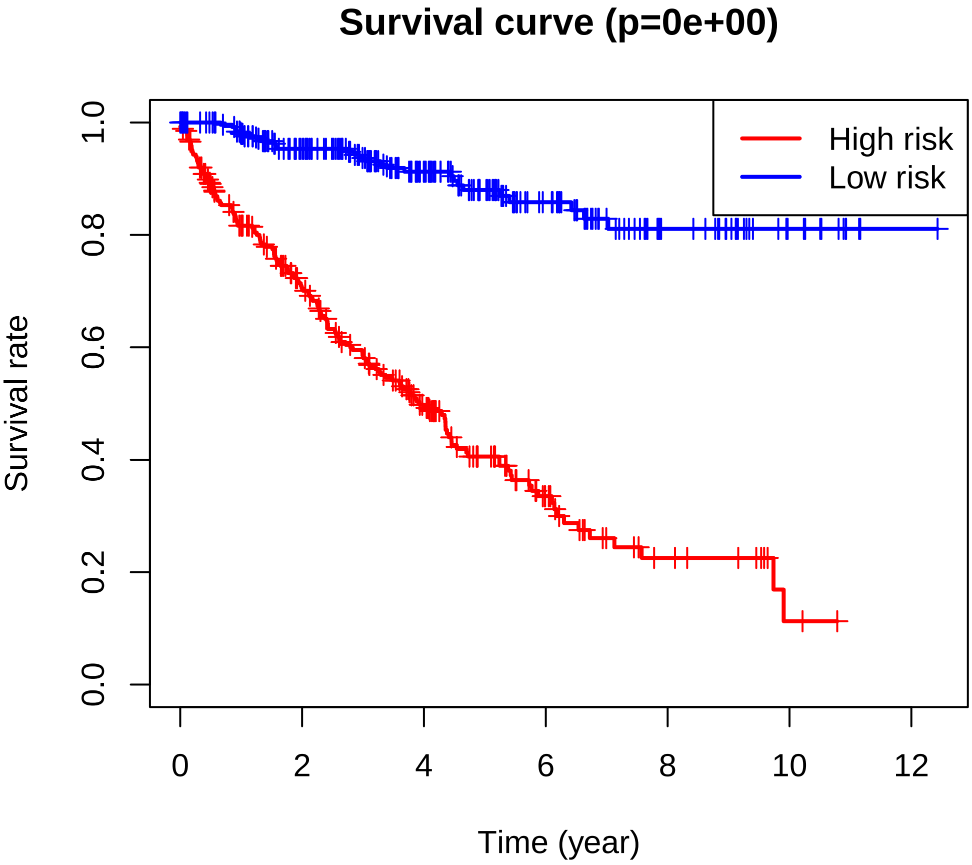
Survival curve (p=0e+00)
High risk
Low risk
Survival rate
0.0
0.2
0.4
0.6
0.8
1.0
0
2
4
6
8
10
12
Time (year)

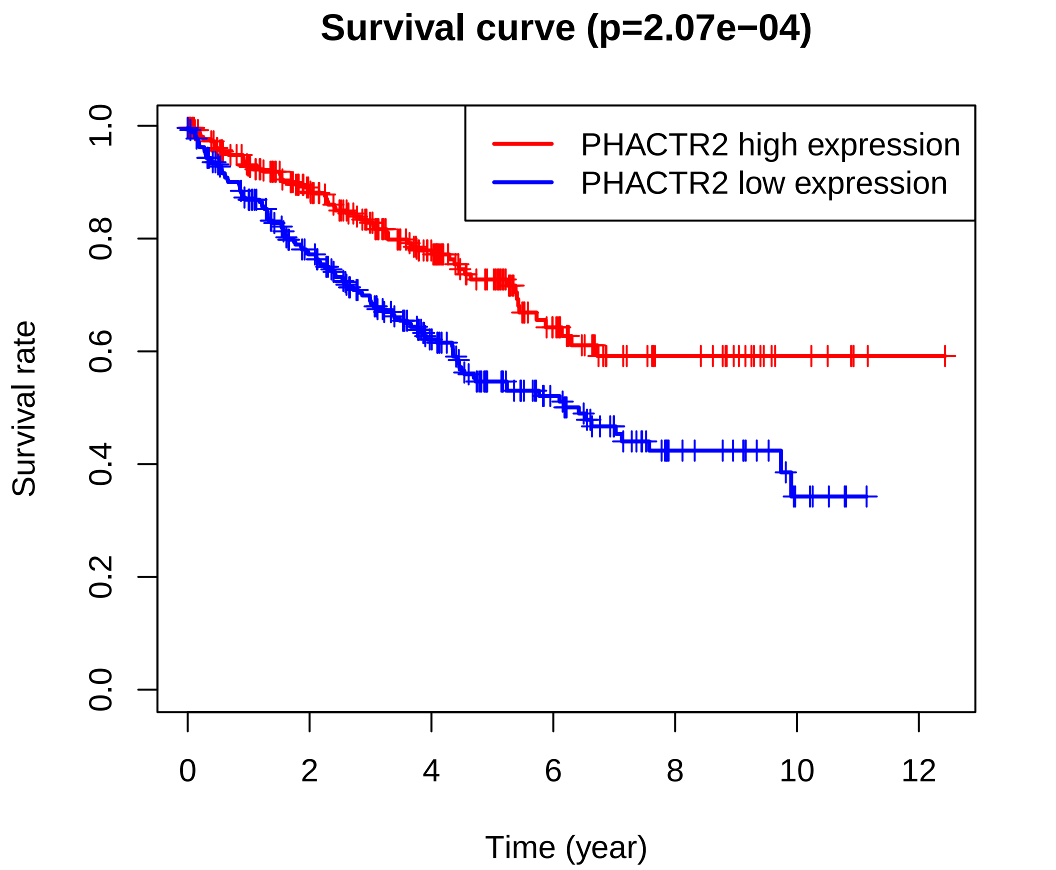

Survival curve (p=2.07e−04)
PHACTR2 high expression
PHACTR2 low expression
Survival rate
1.0
0.8
0.6
0.4
0.2
0.0
0
2
4
6
8
10
12
Time (year)

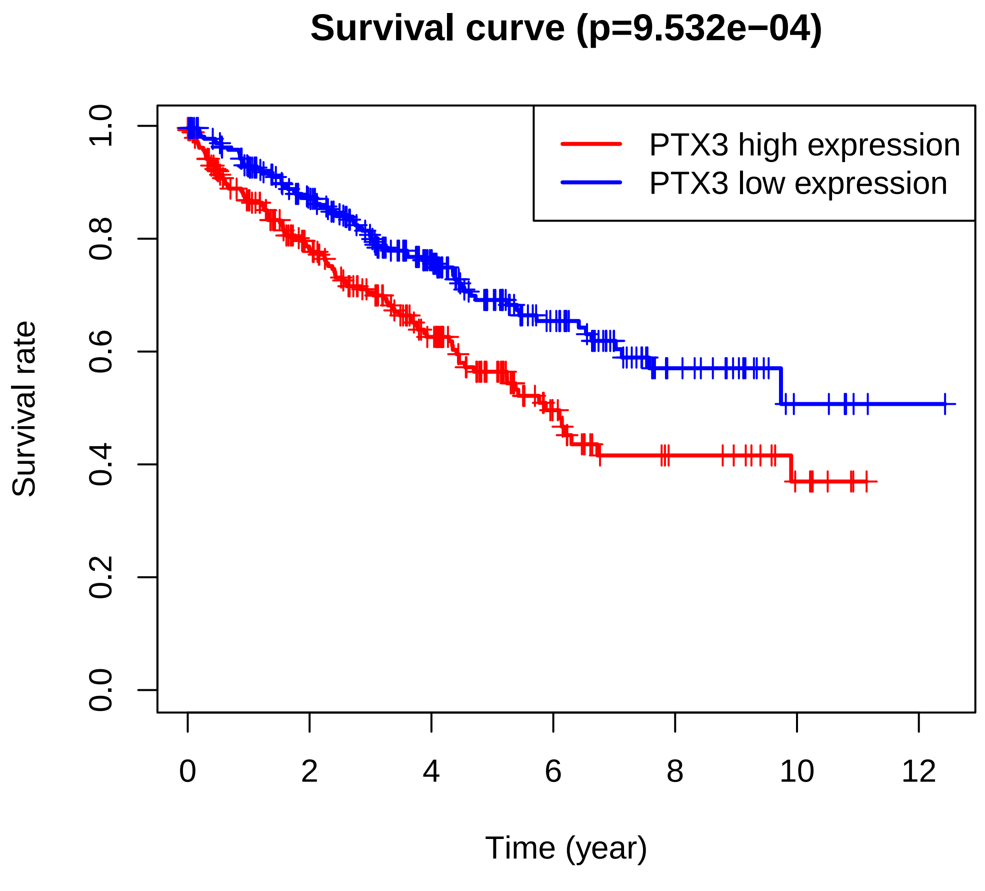

Survival curve (p=9.532e−04)
PTX3 high expression
PTX3 low expression
Survival rate
Time (year)
0.0
0.2
0.4
0.6
0.8
1.0
0
2
4
6
8
10
12

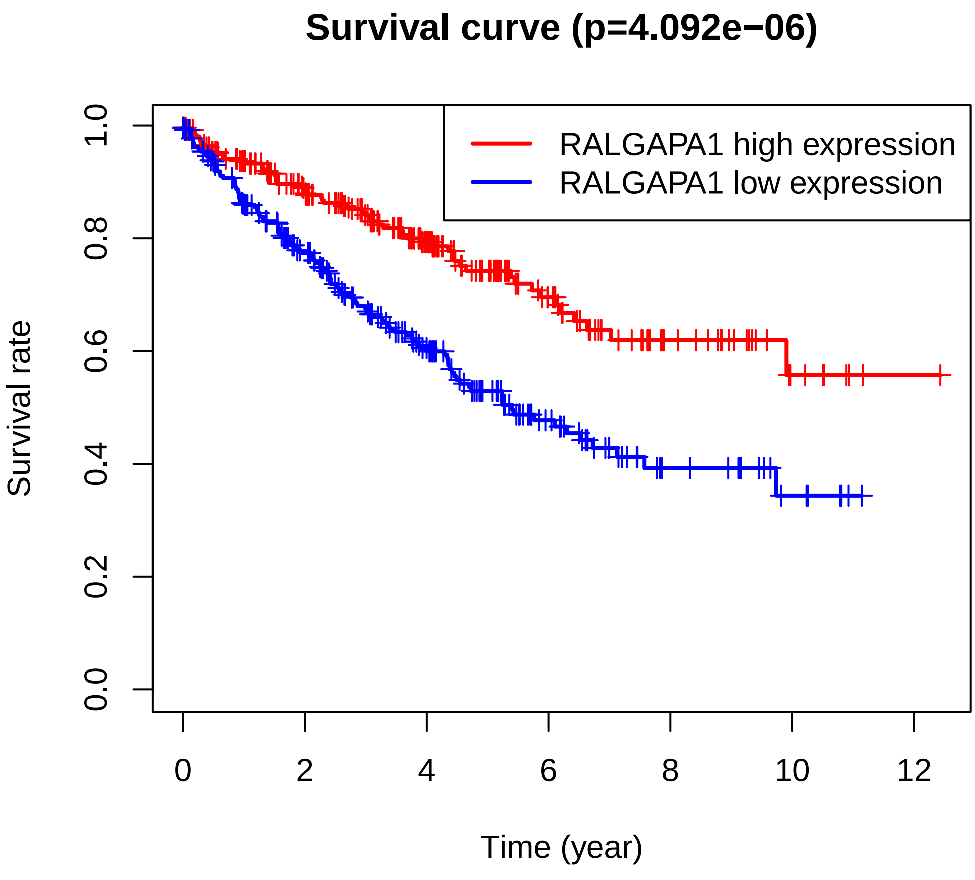

Survival curve (p=4.092e−06)
RALGAPA1 high expression
RALGAPA1 low expression
Survival rate
Time (year)
0.0
0.2
0.4
0.6
0.8
1.0
0
2
4
6
8
10
12

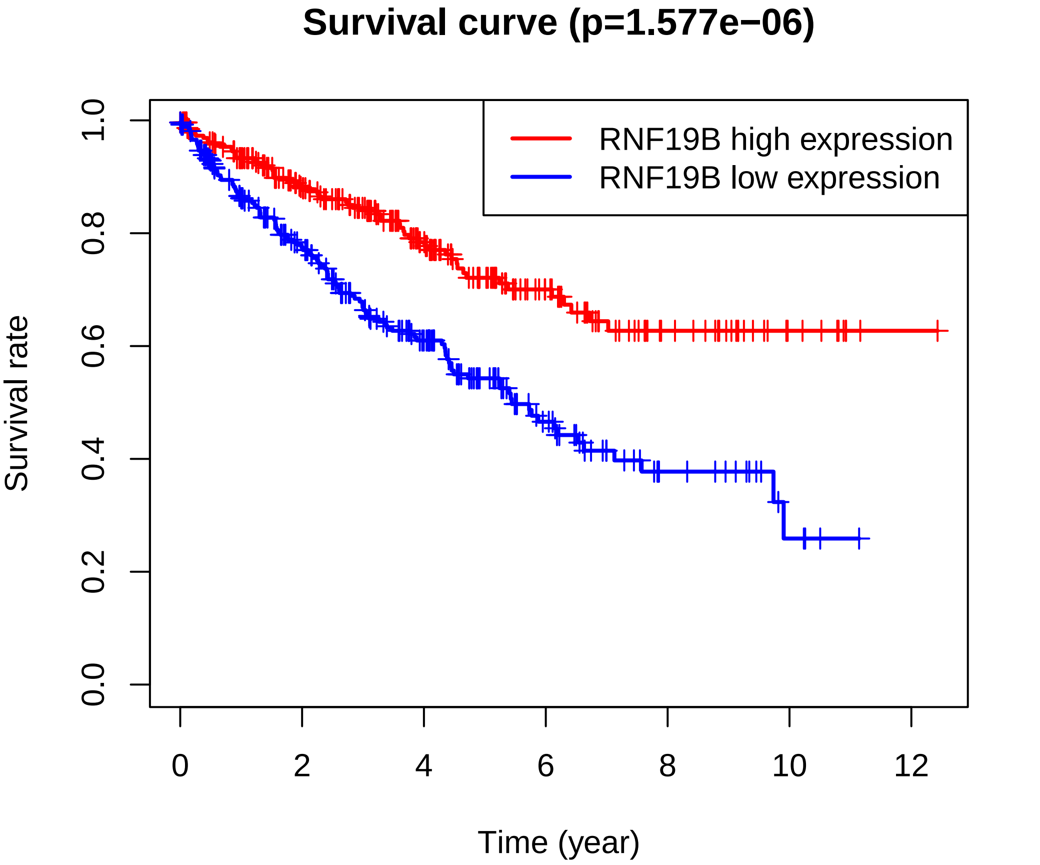

Survival curve (p=1.577e−06)
RNF19B high expression
RNF19B low expression
Survival rate
0.0
0.2
0.4
0.6
0.8
1.0
0
2
4
6
8
10
12
Time (year)

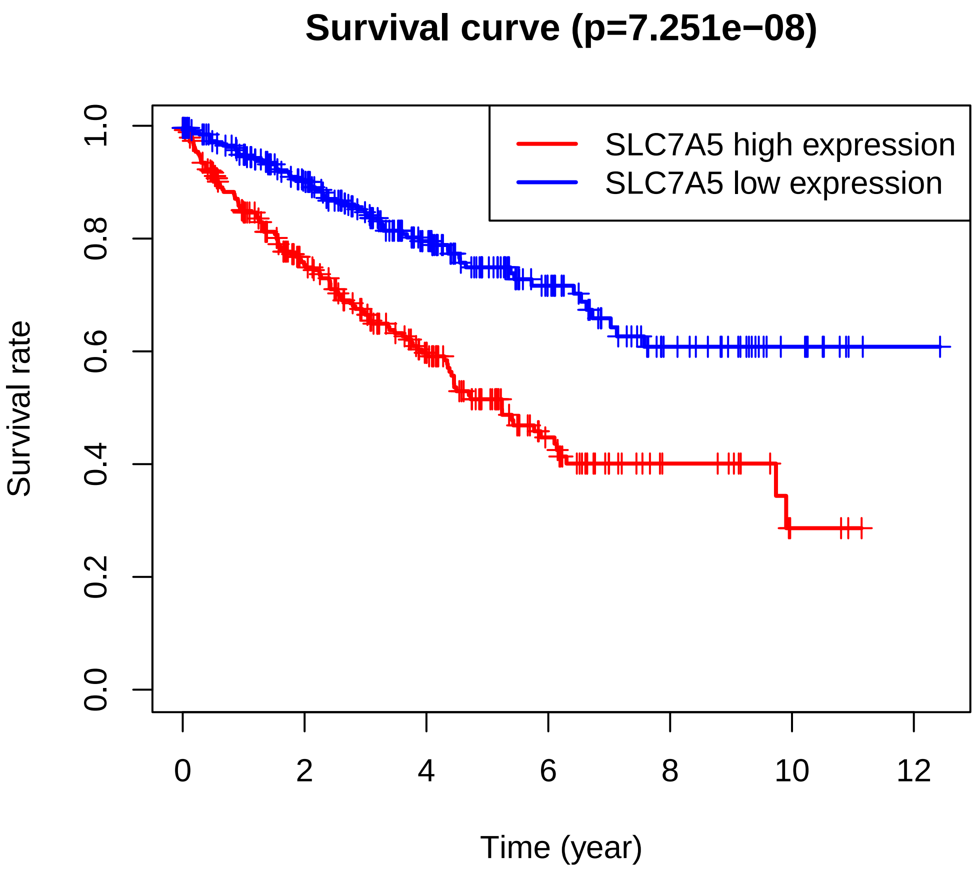

Survival curve (p=7.251e−08)
SLC7A5 high expression
SLC7A5 low expression
Survival rate
0.0
0.2
0.4
0.6
0.8
1.0
0
2
4
6
8
10
12
Time (year)

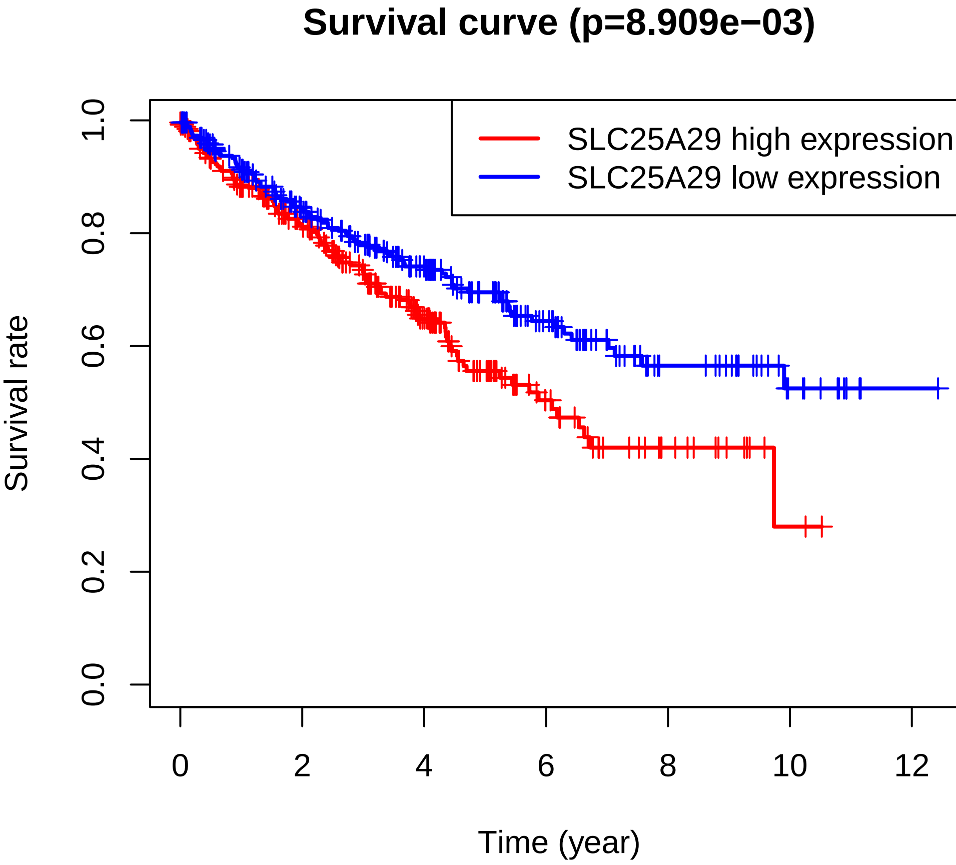

Survival curve (p=8.909e−03)
SLC25A29 high expression
SLC25A29 low expression
Survival rate
Time (year)
0.0
0.2
0.4
0.6
0.8
1.0
0
2
4
6
8
10
12

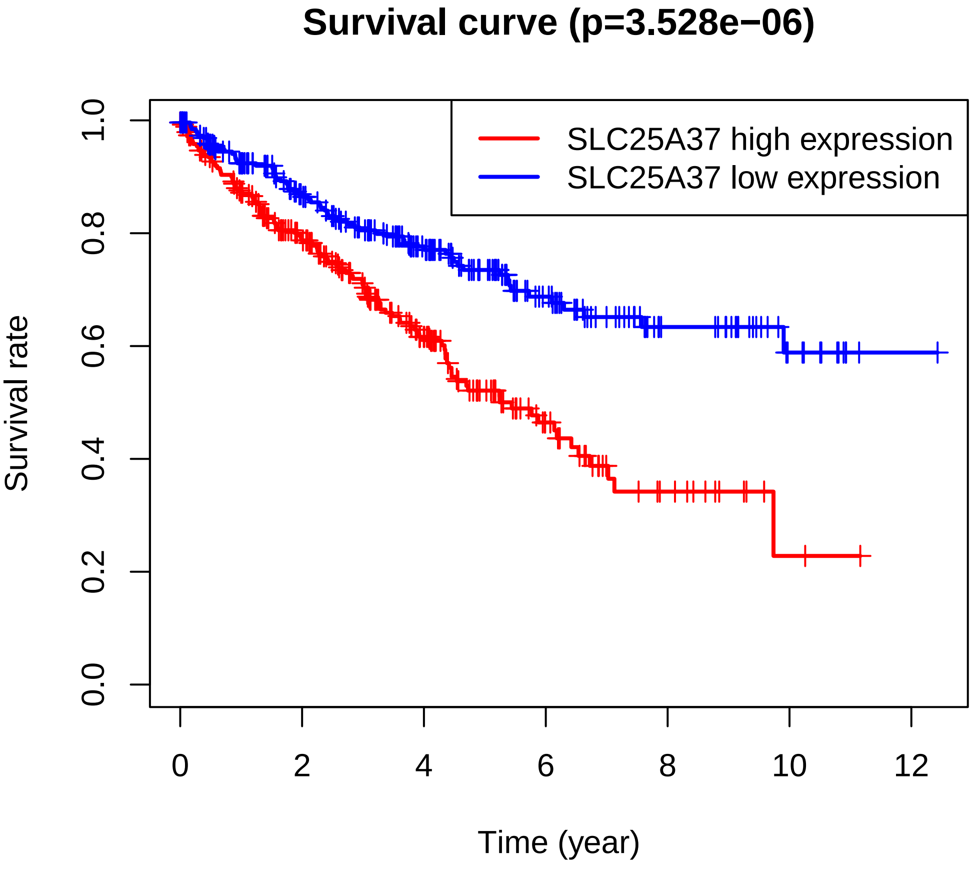

Survival curve (p=3.528e−06)
SLC25A37 high expression
SLC25A37 low expression
Survival rate
0.0
0.2
0.4
0.6
0.8
1.0
0
2
4
6
8
10
12
Time (year)

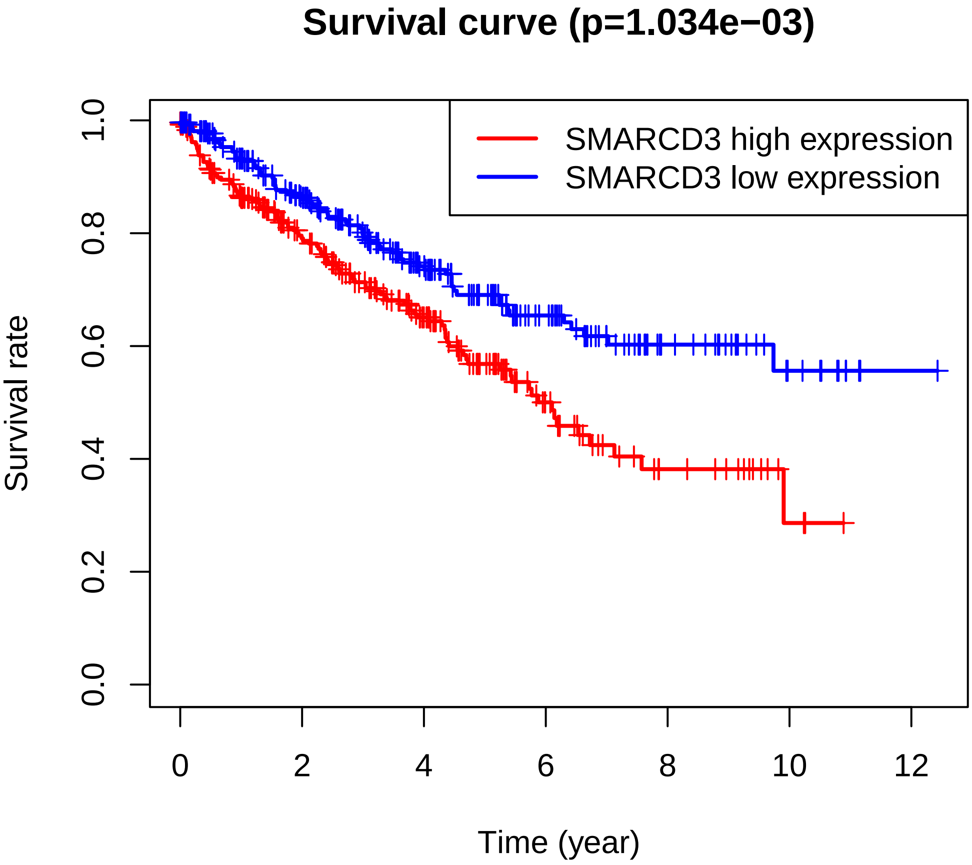

Survival curve (p=1.034e−03)
SMARCD3 high expression
SMARCD3 low expression
Survival rate
1.0
0.8
0.6
0.4
0.2
0.0
0
2
4
6
8
10
12
Time (year)

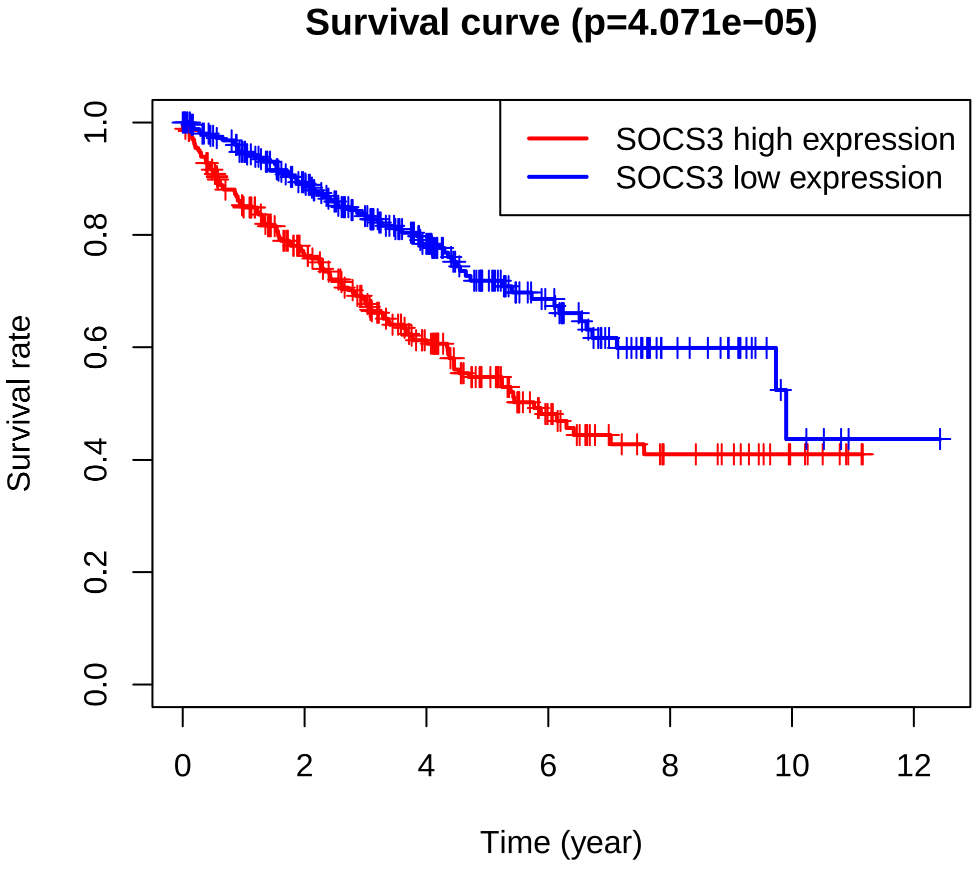
Survival curve (p=4.071e−05)
SOCS3 high expression
SOCS3 low expression
Survival rate
Time (year)
0.0
0.2
0.4
0.6
0.8
1.0
0
2
4
6
8
10
12

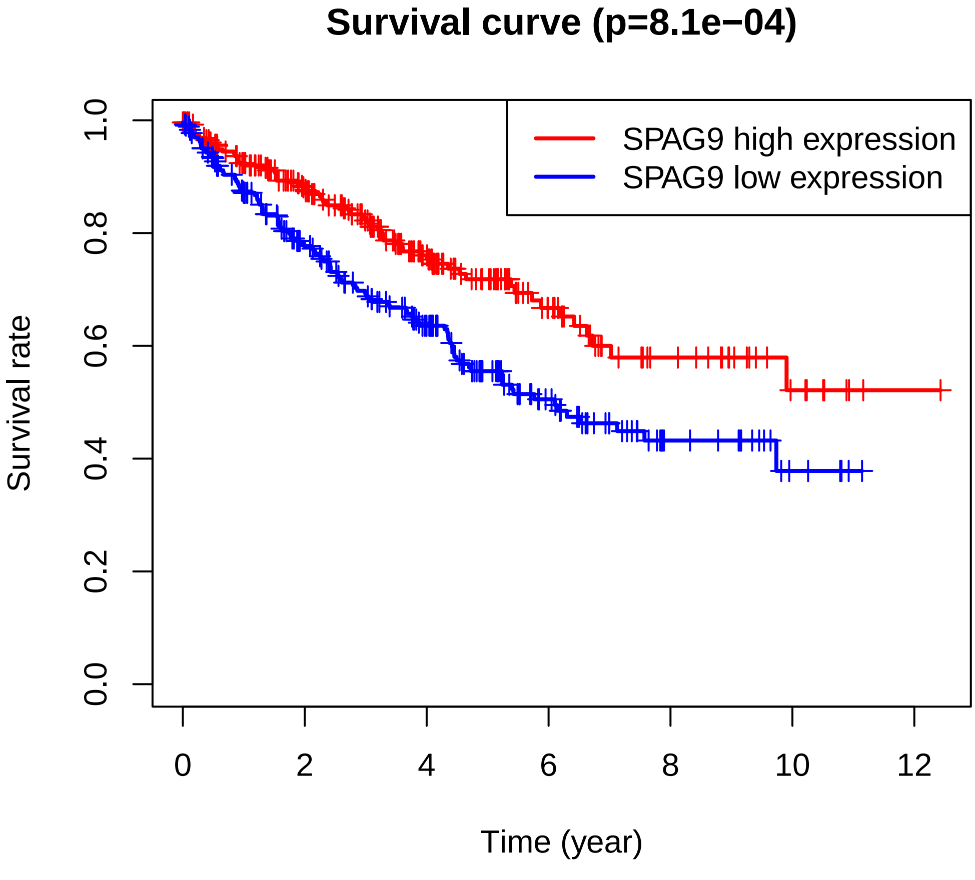

Survival curve (p=8.1e−04)
SPAG9 high expression
SPAG9 low expression
Survival rate
1.0
0.8
0.6
0.4
0.2
0.0
0
2
4
6
8
10
12
Time (year)

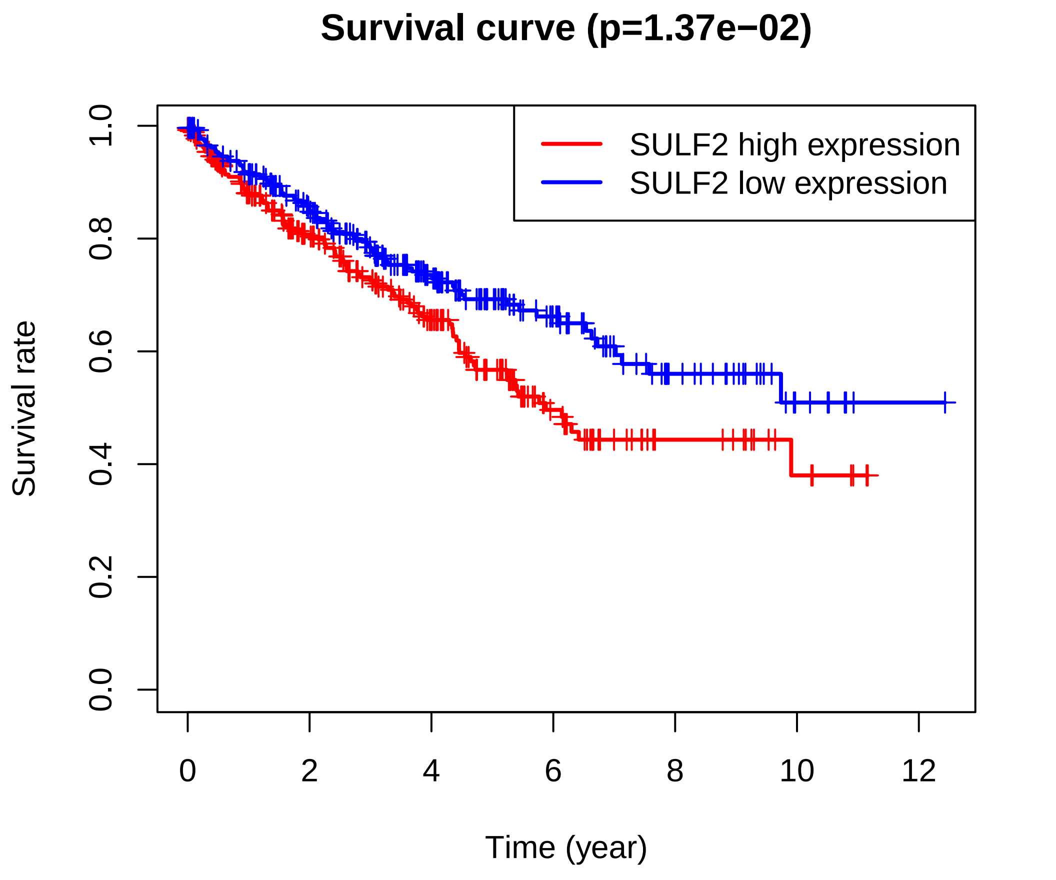

Survival curve (p=1.37e−02)
SULF2 high expression
SULF2 low expression
Survival rate
Time (year)
0.0
0.2
0.4
0.6
0.8
1.0
0
2
4
6
8
10
12

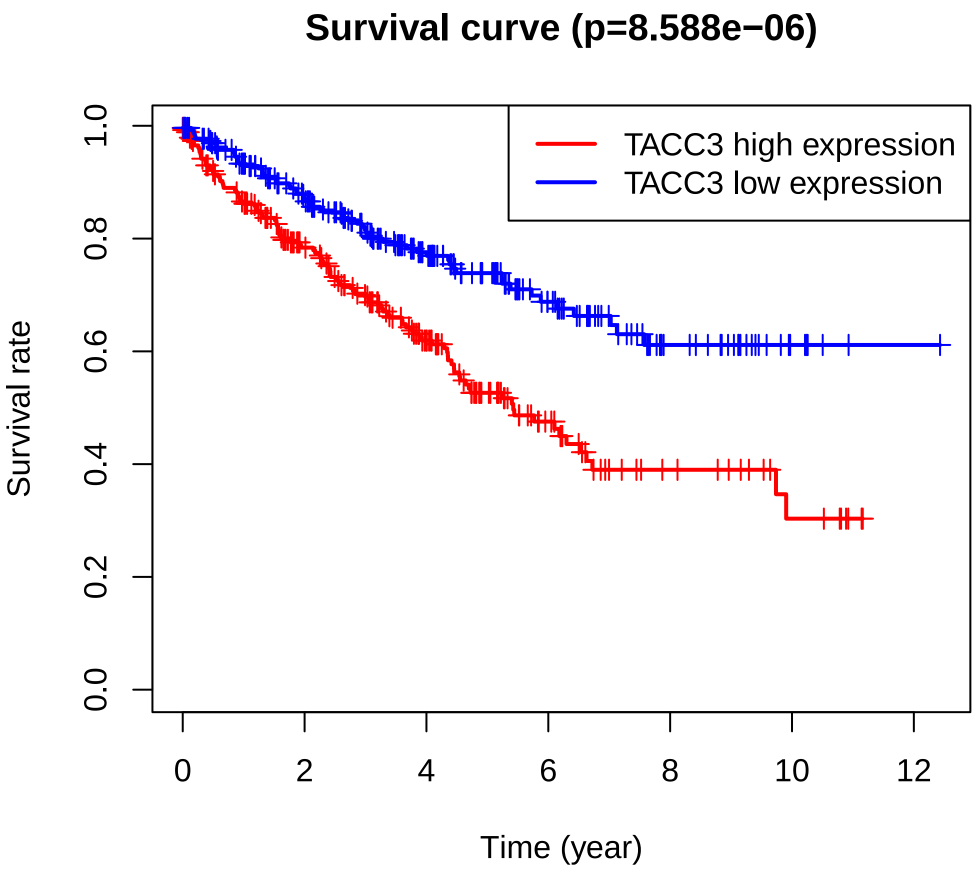

Survival curve (p=8.588e−06)
TACC3 high expression
TACC3 low expression
Survival rate
1.0
0.8
0.6
0.4
0.2
0.0
0
2
4
6
8
10
12
Time (year)

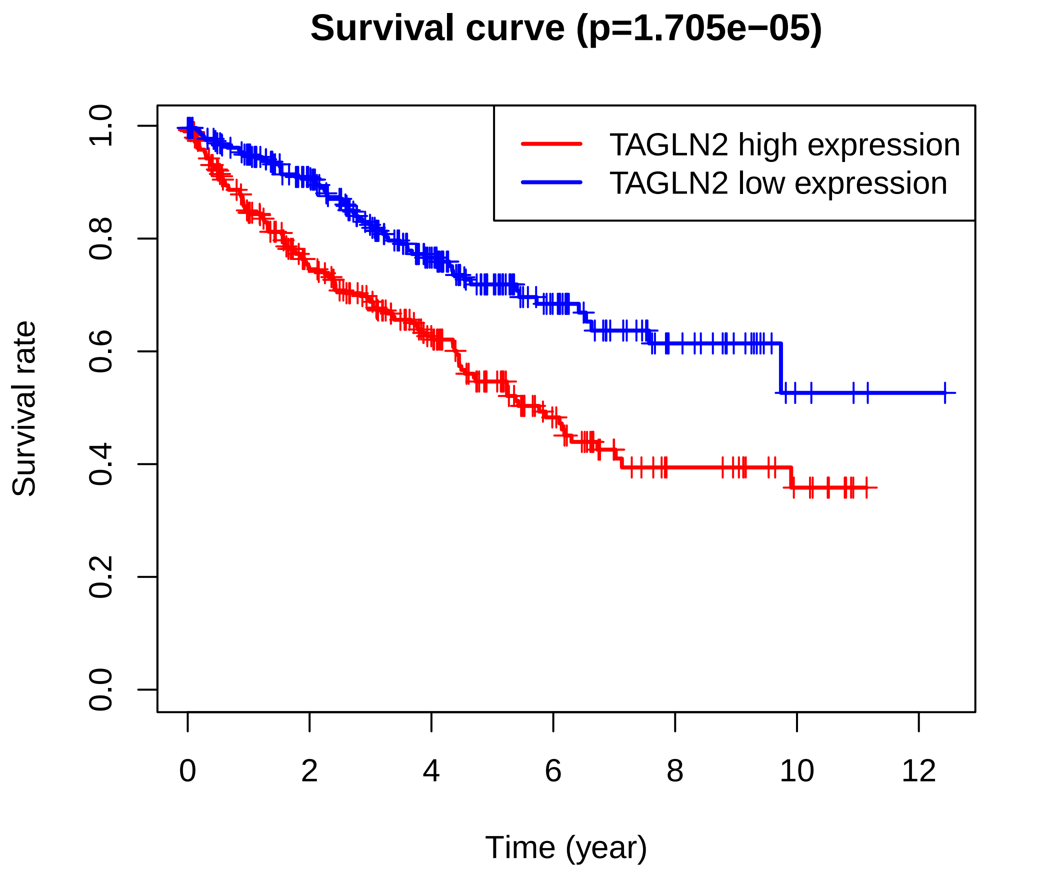
Survival curve (p=1.705e−05)
TAGLN2 high expression
TAGLN2 low expression
Survival rate
1.0
0.8
0.6
0.4
0.2
0.0
0
2
4
6
8
10
12
Time (year)

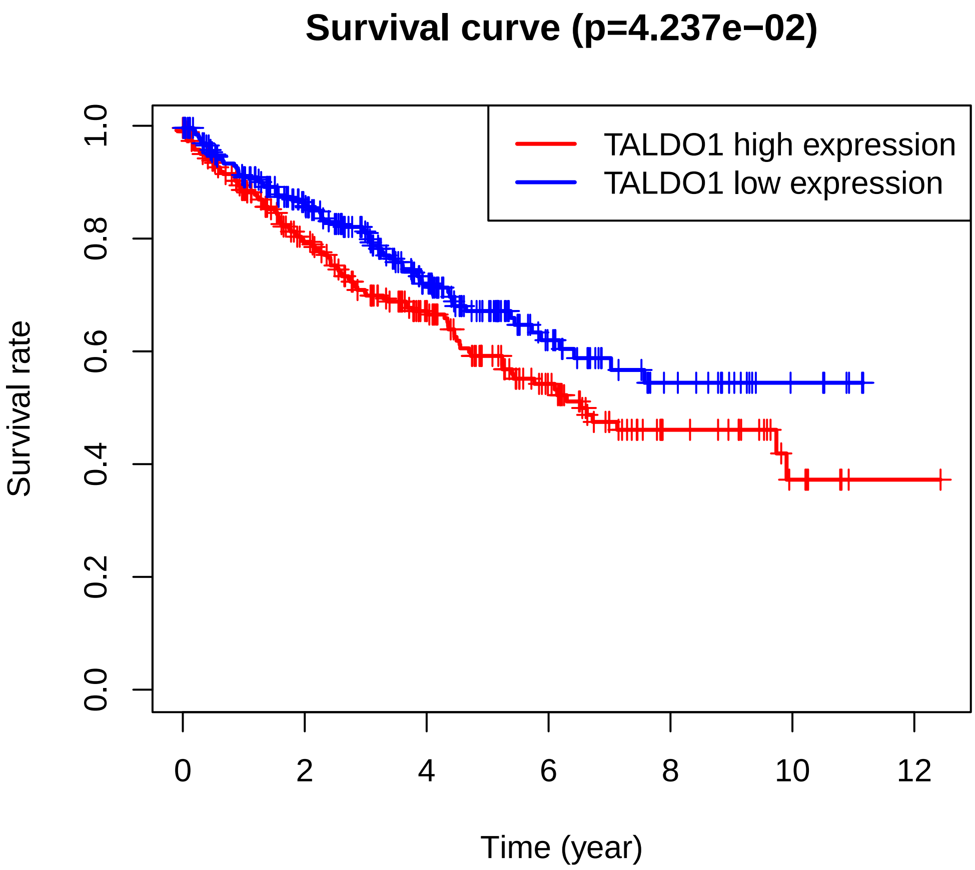

Survival curve (p=4.237e−02)
TALDO1 high expression
TALDO1 low expression
Survival rate
Time (year)
0.0
0.2
0.4
0.6
0.8
1.0
0
2
4
6
8
10
12

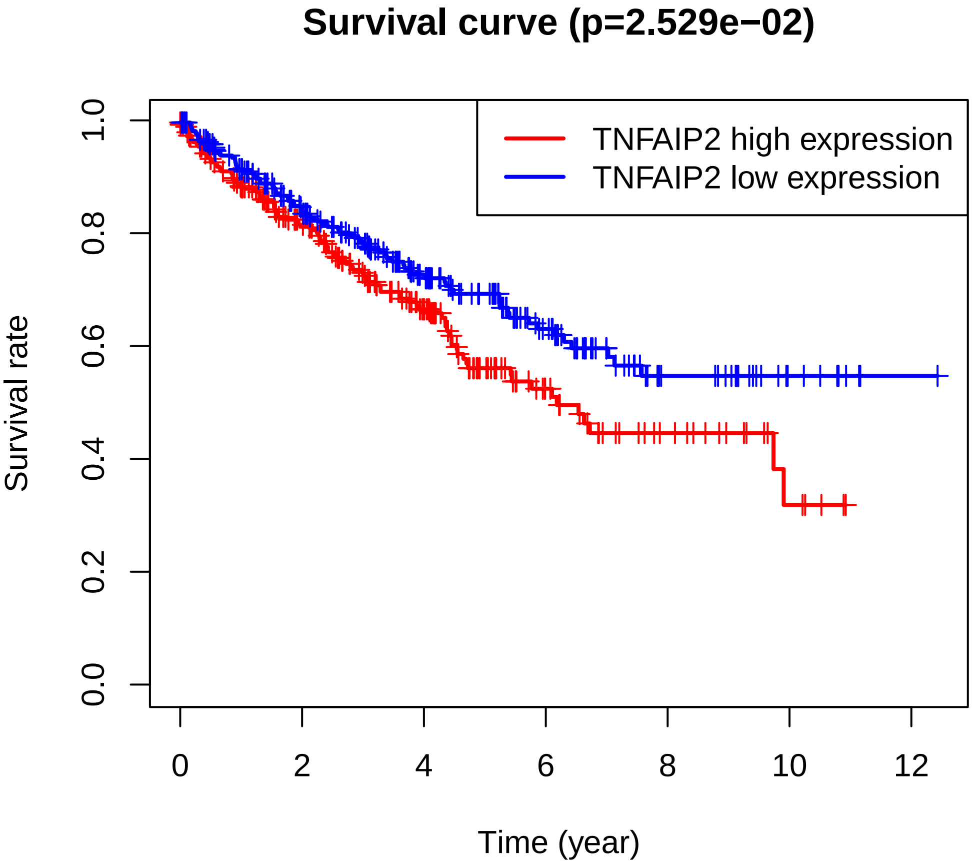
Survival curve (p=2.529e−02)
TNFAIP2 high expression
TNFAIP2 low expression
Survival rate
0.0
0.2
0.4
0.6
0.8
1.0
0
2
4
6
8
10
12
Time (year)

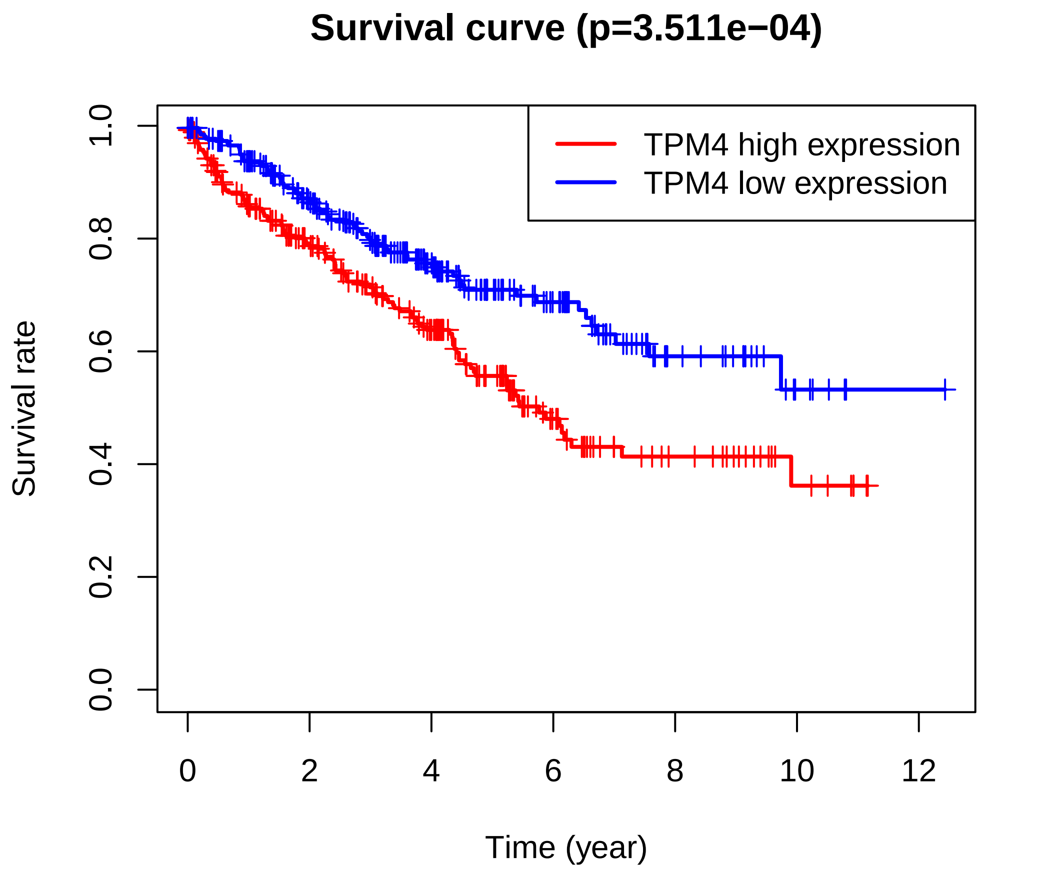
Survival curve (p=3.511e−04)
TPM4 high expression
TPM4 low expression
Survival rate
Time (year)
0.0
0.2
0.4
0.6
0.8
1.0
0
2
4
6
8
10
12

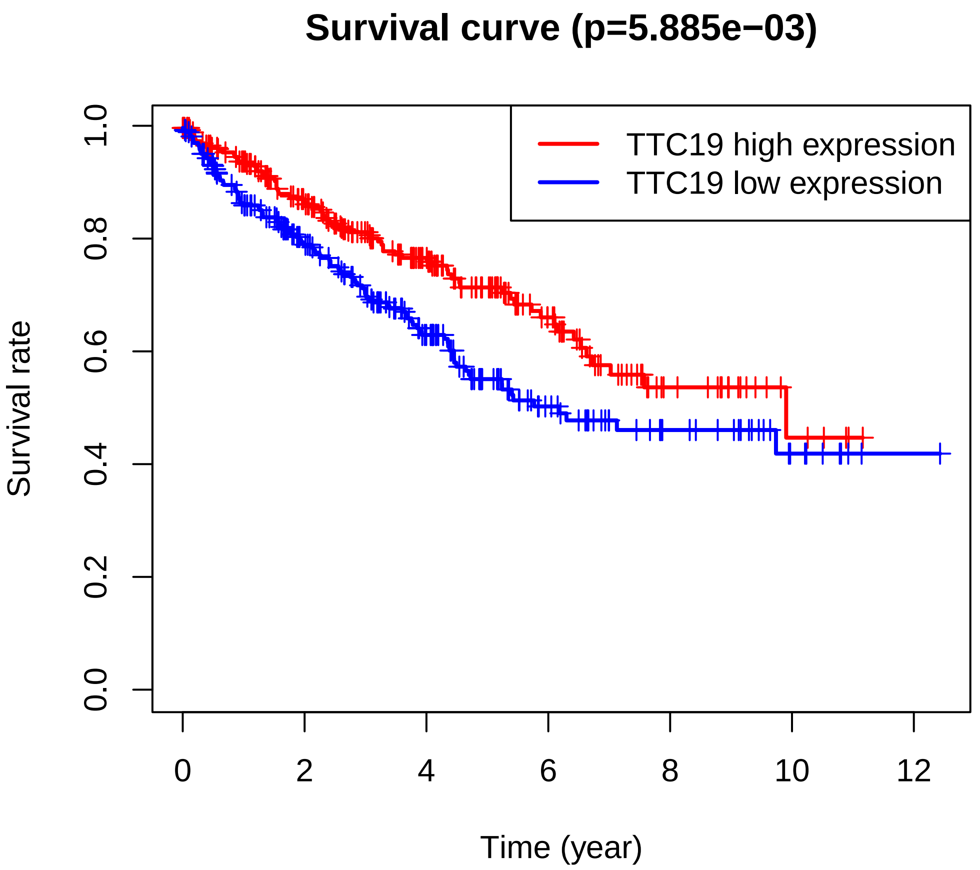

Survival curve (p=5.885e−03)
TTC19 high expression
TTC19 low expression
Survival rate
0.0
0.2
0.4
0.6
0.8
1.0
0
2
4
6
8
10
12
Time (year)

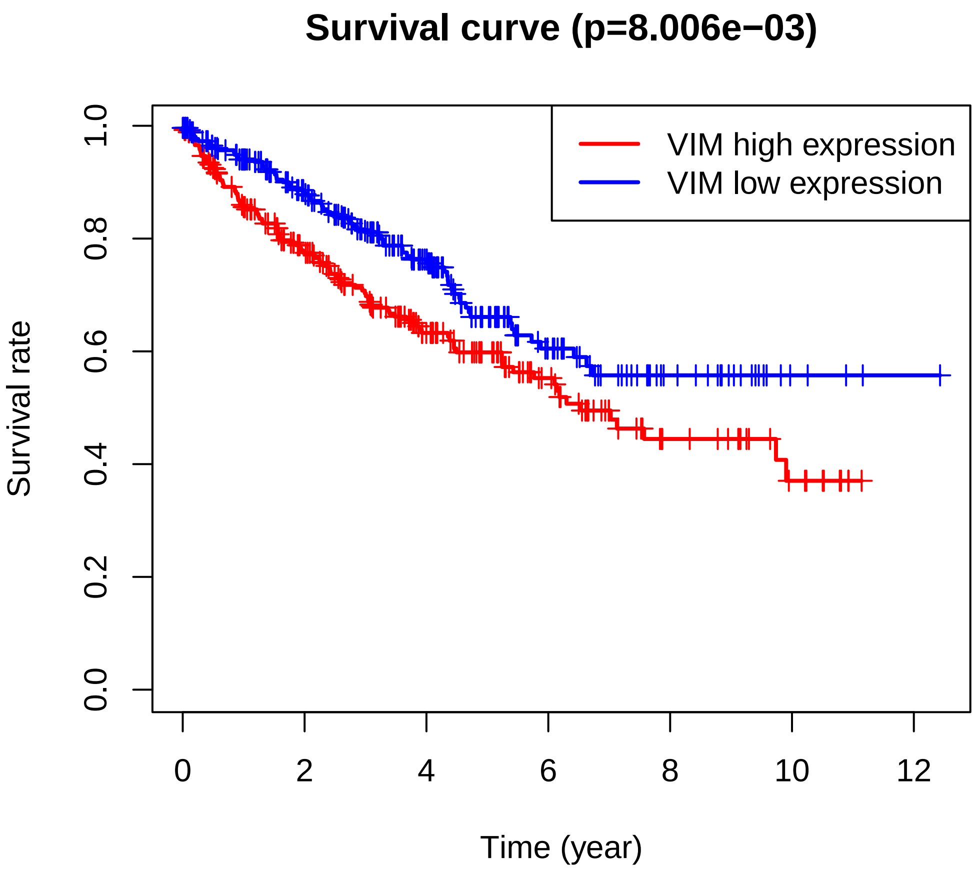

Survival curve (p=8.006e−03)
VIM high expression
VIM low expression
Survival rate
Time (year)
0.0
0.2
0.4
0.6
0.8
1.0
0
2
4
6
8
10
12

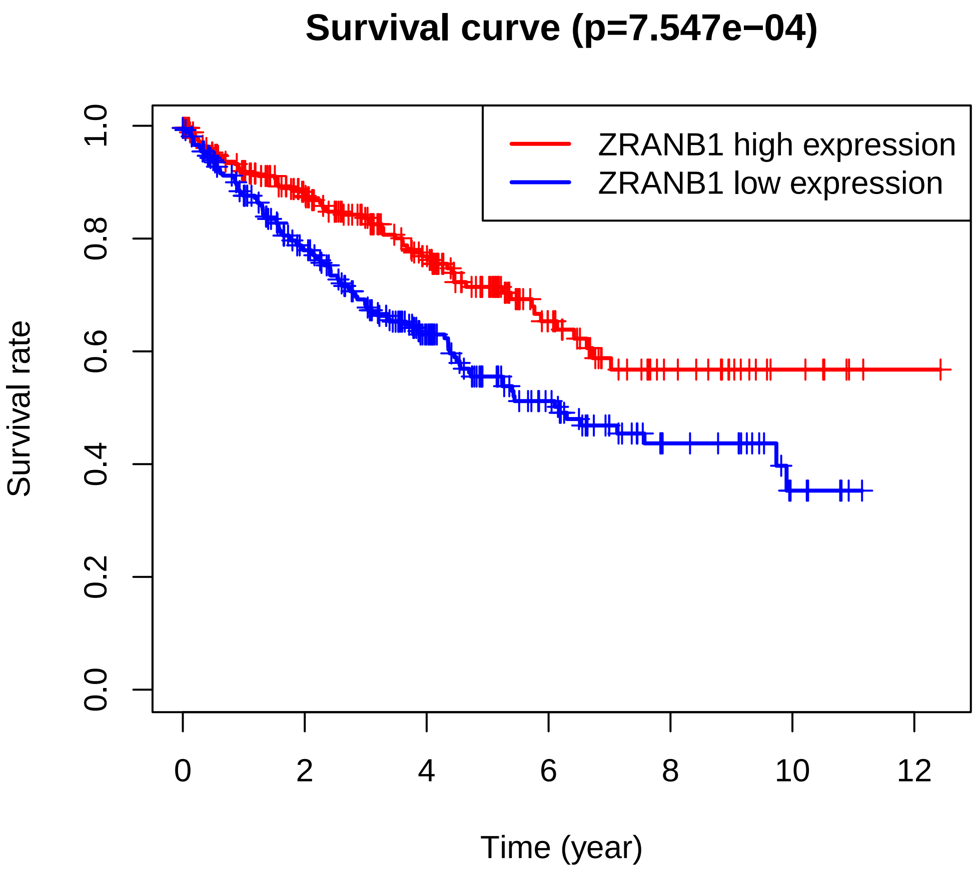
Survival curve (p=7.547e−04)
ZRANB1 high expression
ZRANB1 low expression
Survival rate
Time (year)
0.0
0.2
0.4
0.6
0.8
1.0
0
2
4
6
8
10
12
